# Anti-inflammatory and analgesic effects of Kaihoujian throat spray, and therapeutic mechanism in acute pharyngitis: involvement of the NF-κB/COX-2 pathway and formula deconstruction strategy

**DOI:** 10.3389/fphar.2025.1687046

**Published:** 2025-11-21

**Authors:** Hui Shi, Jinhe Zhang, Liyan Zhang, Xiu Dong, Chang Liu, Xiongwei Liu, Ying Zhou, Tingting Feng

**Affiliations:** 1 School of Pharmacy, Guizhou University of Traditional Chinese Medicine, Guiyang, Guizhou, China; 2 Guizhou Key Laboratory of Modern Traditional Chinese Medicine Creation, Guiyang, Guizhou, China; 3 Guizhou Sanli Pharmaceutical Co., Ltd., Anshun Guizhou, China

**Keywords:** acute pharyngitis, anti-inflammatory and analgesic, NF-κB/COX-2 pathway, ethnopharmacology, HPLC fingerprinting, Kaihoujian (KHJ) throat spray

## Abstract

**Introduction:**

Kaihoujian (KHJ) throat spray is a traditional formula derived from the Miao ethnic minority in China, which is highly effective in treating acute pharyngitis and tonsillitis. This study aimed to investigate the anti-inflammatory and analgesic effects, and therapeutic mechanism, of KHJ in acute pharyngitis. The study hypothesized that the integrated composition of KHJ exerted superior therapeutic effects through synergistic actions.

**Methods:**

The study employed a formula deconstruction strategy, comparing the complete KHJ formula with its individual components. The compatibility of KHJ was assessed through high performance liquid chromatography fingerprinting. Its anti-inflammatory and analgesic properties were evaluated using models of xylene-induced edema, acetic-acid permeability, and hot plate and writhing tests. An acute pharyngitis model was established in rats using 10% ammonia to assess the alterations in behavioral, histological parameters, and serum levels of inflammatory cytokines. Western blot analysis was performed to analyze the expression of cyclooxygenase-2 (COX-2) and nuclear factor kappa B (NF-κB) pathway proteins. Molecular docking was utilized to investigate the interactions between bergenin, matrine, oxymatrine, and macaine of KHJ and inflammatory targets COX-2, NF-κB p65, and p-NF-κB p65.

**Results:**

The fingerprint and assay results indicated no significant changes in the number of chromatographic peaks of KHJ before and after compatibility, though the elution of indicator components did interact. KHJ’s whole formula outperformed separated components in anti-inflammatory and analgesic assays. In pharyngitis, KHJ reduced pathological damage, downregulated interleukin 1β (IL-1β), interleukin 6 (IL-6), prostaglandin E2 (PGE2), and elevated interleukin 10 (IL-10). Western blot revealed KHJ suppressed COX-2 and p-NF-κB p65/NF-κB p65 expression. Molecular docking supported strong binding affinities between KHJ’s active compounds and inflammatory targets.

**Conclusion:**

This study confirmed the potent anti-inflammatory and analgesic effects of the Miao medicine KHJ and its efficacy in treating acute pharyngitis. It was novel in reporting the complete KHJ formula as significantly superior to its individual components, thereby underscoring the vital role of integrated formulation design. Furthermore, the therapeutic effect was mechanistically associated with inhibition of the COX-2/NF-κB signaling pathway. These findings provide a scientific foundation for the clinical application of KHJ and validate the rationale of Miao medicine compatibility.

## 1 Introduction

Acute pharyngitis is a prevalent upper respiratory tract disorder characterized by pharyngeal redness, swelling, pain, and inflammatory reactions, significantly impairing the quality of life of patients (Jiang et al., 2016). The primary pathogenesis involves inflammatory responses triggered by viral or bacterial infections and physicochemical irritants (Gu et al., 2025). If not timely treated, recurrent acute pharyngitis may progress to chronic disease, further complicating treatment (Ding et al., 2020). Currently, non-steroidal anti-inflammatory drugs are widely used in clinical practice to treat various types of pain and inflammation-induced fever (Huang et al., 2024; Huynh et al., 2023). However, the prolonged use of these agents is associated with adverse effects, including antibiotic resistance and gastrointestinal complications (Muzio et al., 2016), thus highlighting the need for safer and more effective therapeutic alternatives.

Traditional Chinese medicine (TCM) offers a promising approach due to its multi-component, multi-target mechanisms. It has synergistic anti-inflammatory and analgesic properties with fewer side effects. Among TCM formulations, Kaihoujian (KHJ) throat spray has been widely used for treating throat disorders, demonstrating notable efficacy in alleviating inflammation and pain. KHJ is formulated with *Ardisia crenata* Sims (A), *Sophorae tonkinensis* Gagnep (S), *Cryptotympana pustulata* Fabricius (C), and L-Menthol (M). KHJ is particularly effective in treating acute pharyngitis, stomatitis, oral ulcers, and swollen and painful gums, as well as herpetic stomatitis, herpangina, acute suppurative tonsillitis, hand-foot-mouth disease, and thrush (Pang et al., 2023; Ma et al., 2021).

KHJ is a traditional Miao medicine formula in China. It has been suggested that Miao medicine prescriptions typically comprise three fundamental components: main medicine (Zhuyao), auxiliary medicine (Fuyao), and guide medicine (Yinyao) (Du, 2006). This approach is analogous to the principle of “Jun–Chen–Zuo–Shi” in TCM. According to Miao medicine theory, the main medicine in KHJ is *A. crenata* Sims (A), which is cold in nature and belongs to the hot meridian. It is known for its throat-clearing, blood-stasis-removing, and swelling-reducing effects (Chinese Pharmacopoeia Commission., 2020a). Its primary metabolite, bergenin, is recognized for its strong anti-inflammatory properties (Tang et al., 2021). *Sophorae tonkinensis* Gagnep. (S) serves as the auxiliary medicine and combines with *A. crenata* Sims (A) to form the “main medicine composition”. It is also cold in nature, associated with the hot meridians, and has heat-clearing, detoxifying, edema-relieving, and sore throat-alleviating effects (Chinese Pharmacopoeia Commission, 2020b). Its active metabolites, matrine and oxymatrine, exhibit significant anti-inflammatory effects (Li et al., 2021; Wang et al., 2021). L-Menthol (M) is extracted from the leaves and rhizomes of peppermint in the form of white crystals (Chinese Pharmacopoeia Commission., 2020c). It supports the entire formula, has a cold nature, belongs to the hot meridian, and has relaxing, antibacterial, and anti-inflammatory activities (Nagai et al., 2023). *Cryptotympana pustulata* Fabricius (C) is used as a guide medicine. It is a heat-clearing botanical drug that can disperse wind and clear heat (Chinese Pharmacopoeia Commission., 2020d). Its effective metabolites, including proteins, amino acids, and chitin, enhance immune function and promote self-healing in the body (Mei et al., 2023). The combination of M and C as auxiliary medicines enhances the overall efficacy of the prescription. Consequently, the synergistic use of these components in the KHJ formula effectively clears heat, detoxifies the body, reduces swelling, and relieves pain. KHJ exhibits remarkable therapeutic efficacy with high safety and minimal side effects in treating acute pharyngitis and tonsillitis (Ma et al., 2021). Despite this, studies on the rationale behind the compatibility of KHJ and its anti-inflammatory and analgesic mechanisms in treating acute pharyngitis are scarce. This study involved a formula deconstruction of KHJ based on Miao medicine theory, with the following experimental groups: complete formula group of Kaihoujian (KHJ), main medicine group of *A. crenata* Sims (A), main and auxiliary medicine group of *A. crenata* Sims and *Sophorae tonkinensis* Gagnep (AS), and auxiliary and guide medicine group of L-Menthol and *C. pustulata* Fabricius (MC).

**FIGURE 1:**
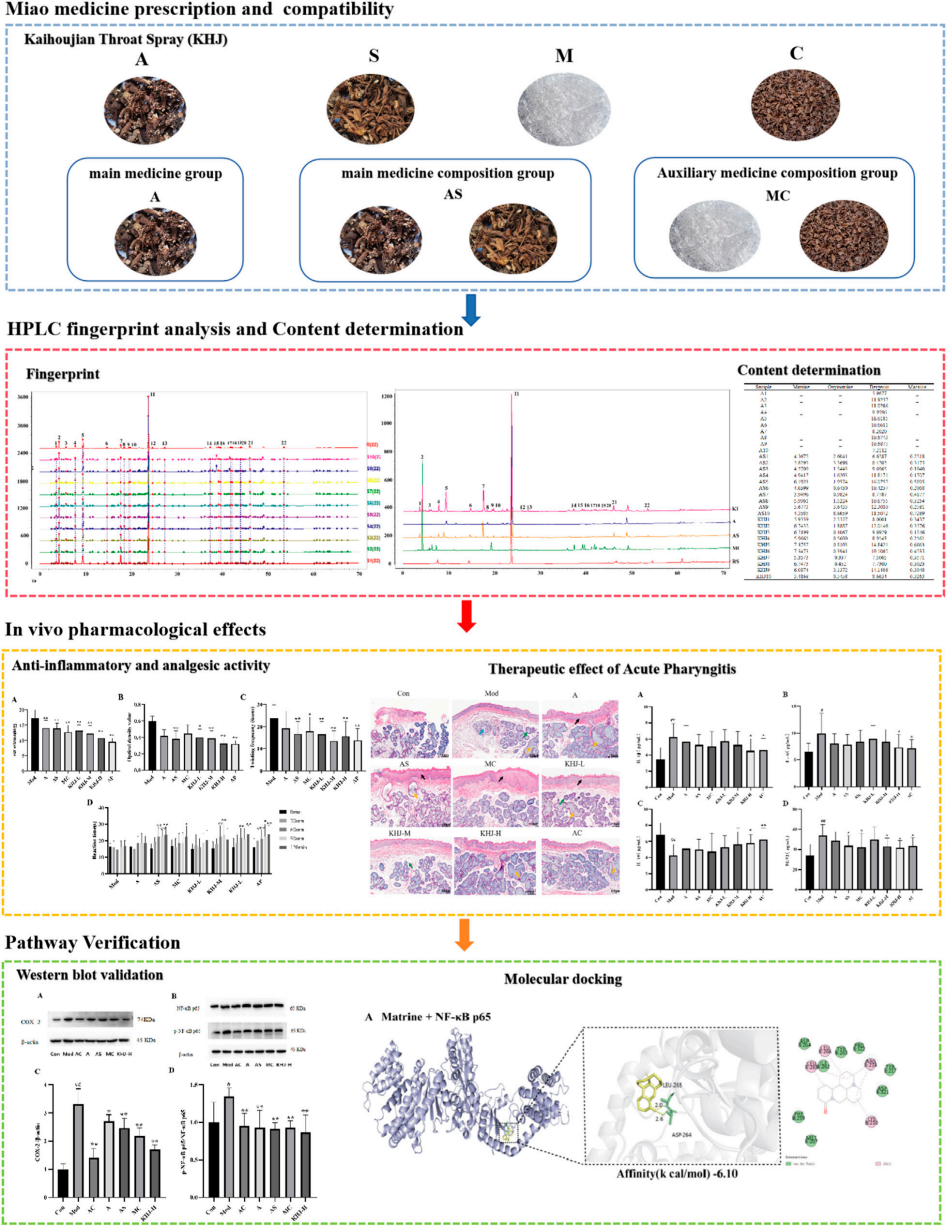
Overall flowchart of this study.

The present study was conducted to systematically investigate the anti-inflammatory and analgesic properties of the Miao medicine KHJ, its therapeutic efficacy against acute pharyngitis, and its underlying mechanisms by applying a comprehensive formula deconstruction approach. A high-performance liquid chromatography (HPLC)-based analytical method was used to examine the variations in fingerprint spectrum and the content of components before and after formula combination. The anti-inflammatory and analgesic activities were assessed using multiple experimental models, including xylene-induced mouse ear edema, acetic acid-induced capillary permeability, hot plate tests, and acetic acid-induced writhing responses, to compare the complete formula with its individual components. Furthermore, a rat model of acute pharyngitis was established to evaluate the therapeutic efficacy and explore the underlying mechanisms of KHJ. This study was the first formula deconstruction study of KHJ, providing direct evidence for the crucial role of the complete formula in exerting optimal therapeutic effects. The results demonstrated the NF-κB/COX-2 signaling pathway as the core mechanism mediating the anti-pharyngitis effects of KHJ. Molecular docking analysis further supported the interactions between KHJ components and key targets in this pathway. These findings establish a solid foundation for further exploring the therapeutic mechanisms of KHJ and provide scientific validation for the combinatorial principle of Miao medicine formulas (Figure 1).

## 2 Materials and methods

### 2.1 Chemicals and reagents

Matrine (PS011495), oxymatrine (PS011510), bergenin (PS010520), and macaine (PS210802-06) were purchased from Chengdu Pusi Biotechnology Co., Ltd. (Sichuan, China), with purity greater than 98%. The IL-1β, IL-6, IL-10, and PGE2 enzyme-linked immunosorbent assay (ELISA) kits were acquired from Shanghai Zhuocai Biotechnology Co., Ltd. (Shanghai, China). COX-2, NF-κB p65, and p-NF-κB p65 antibodies were obtained from Cell Signaling Technology (MA, United States). Aspirin enteric-coated tablets were purchased from Bayer Healthcare Co., Ltd. (Leverkusen, Germany), and amoxicillin capsules were obtained from Hainan Xiansheng Pharmaceutical Co., Ltd. (Hainan, China).

### 2.2 Plant materials

The medicinal materials in the prescription were provided by Guizhou Sanli Pharmaceutical Co., Ltd. (Guizhou, China), and identified by Professor Wei Shenghua of Guizhou University of Traditional Chinese Medicine. A is the dried root of *A. crenata* Sims, a plant belonging to the family Ardisiaceae. S is the dried root and rhizome of the leguminous plant *Sophora tonkinensis* Gagnep. C is the shed shell of the black grasshopper *Cryptotympanaatrata (*Fabricius) belonging to the family Cicadidae. M is a saturated cyclic alcohol extracted from the fresh stems and leaves of *Mentha haplocalyx* Briq., belonging to the family Lamiaceae, through steam distillation, freezing, and recrystallization. Its chemical structure is l-1-methyl-4-isopropylcyclohexanol-3 (L-Menthol). The source and batch information of medicinal material samples are displayed in Supplementary Table S1. The samples were meticulously preserved in the Guizhou Key Laboratory of Modern Traditional Chinese Medicine Creation at Guizhou University of Traditional Chinese Medicine.

### 2.3 Preparation of extracts

After preparing KHJ, 313 g of A, 313 g of S, and 250 g of C were taken. The mixture was then decocted twice for 2 and 1 h. After filtering, the decoction solution was combined and concentrated into a clear paste with a relative density of 1.05–1.10 (at 50 °C) at 70 °C–80 °C. Ethanol was added to achieve an alcohol content of 80%. The mixture was allowed to stand for 24 h and filtered, followed by the addition of 1 g of L-menthol (M). Ethanol was recovered under reduced pressure. The resulting mixture was freeze-dried under vacuum using a freeze dryer. The powder yield was calculated. According to the theory of Miao medicine prescriptions, the extracts of A, AS, and MC were prepared following the proportions and preparation processes specified in the KHJ prescription. The freeze-dried powder was then stored in a dryer.

### 2.4 HPLC fingerprint analysis and content determination

#### 2.4.1 Chromatographic conditions

HPLC analysis was conducted using an Agilent 1260 system (Agilent Technologies, United States). The chromatographic separations were performed on a DIKMA Platisil-ODS column (250 × 4.6 mm, 5 µm). The mobile phase consisted of methanol (A) and a solution of 0.05% phosphoric acid and 0.1% triethylamine (B). The gradient elution program was as follows: 0–8 min, 10% A; 8–19 min, 10%–30% A; 19–28 min, 30%–30%A; 28–32 min, 30%–40% A; 32–40 min, 40%–60% A; 40–70 min, 60%–100% A. The flow rate was 0.8 mL/min, and the column temperature was maintained at 35 °C. The injection volume was 10 μL, and the detection wavelength was set to 220 nm.

#### 2.4.2 Preparation of standard solutions

Appropriate amounts of bergenin, matrine, oxymatrine, and macaine were accurately weighed to prepare stock solutions with concentrations of 2.094, 1.017, 1.034, and 0.416 mg/mL, respectively.

#### 2.4.3 Preparation of test solutions for HPLC

Further, 0.1 g of KHJ freeze-dried powder was accurately weighed and placed in a conical flask with a cover. Then, 25 mL of 25% methanol was added, and the flask was weighed. The mixture was sonicated for 5 min, allowed to cool, reweighed, and shaken. The solution was filtered through a 0.22 μm microporous membrane to obtain the test solution for the complete formula. The test solutions from ten batches of the KHJ group were designated as S1–S10.

### 2.5 Experimental animals

A total of 96 specific pathogen free (SPF)-grade male Kun Ming mice, each weighing 20 ± 2 g, were obtained from Changsha Tianqin Biotechnology Co., Ltd., under license number SCXK (Xiang) 2019-0013. The study was reviewed and approved by the Experimental Animal Ethics Committee of Guizhou University of Chinese Medicine (No. 20220008). Additionally, 108 SPF-grade Sprague-Dawley rats (54 male and 54 female, weighing 180–220 g) were provided by Beijing Huafu Kang Biotechnology Co., Ltd., under license number SCXK (Jing) 2019-0008. This study was reviewed and approved by the Experimental Animal Ethics Committee of Guizhou University of Chinese Medicine (No. 20210064). During the experiments, the rats were housed in the laboratory animal room at Guizhou University of Traditional Chinese Medicine, which was maintained at a temperature of 22 °C ± 2 °C and a humidity level of 50% ± 10%.

### 2.6 Grouping and administration of anti-inflammatory and analgesic activity

Following a period of adaptive feeding, the mice were randomly assigned to one of the following groups (*n* = 12): Mod group, A group, AS group, MC group, KHJ-L group, KHJ-M group, KHJ-H group, and positive drug AP group. The KHJ-H dosage was determined to be 22 g/kg based on the maximum clinical dose of KHJ and the equivalent dose ratio for mice, as well as previous studies on the maximum tolerated dose in mice (Xiang and Yuan, 2019). The doses for the A, AS, and MC groups were calculated as 7.85, 15.7, and 6.3 g/kg, respectively, based on the formula ratio of the spray. AP was administered to rats in the positive control group at a dose of 0.20 g/kg.

### 2.7 Anti-inflammatory activity

#### 2.7.1 Xylene-induced ear edema

The mice were administered different concentrations of the drug solutions intragastrically at a fixed time each morning for seven consecutive days. The mice in the Mod group received distilled water, whereas those in the other groups were treated with the appropriate doses of the test solutions. Next, 20 μL of xylene solution was applied to the front and back of the right auricle of each mouse 1 h after the final administration, leaving the left auricle untreated as a self-control. The mice were euthanized after 0.5 h, and the left and right ears were excised. The auricles were removed using a punch, and weighed. The degree of swelling and inhibition rate were calculated (Jin et al., 2024; Xie et al., 2023) as follows:

$$\text{Degree of swelling }\left(\text{mg}\right)=\text{Weight of right ear }\left(\text{mg}\right)-\text{Weight of left ear }\left(\text{mg}\right)$$

$$\text{Inhibition rate }\left(\%\right)=\left[\left(\text{Average swelling degree of mice in the }\mathrm{Mod}\text{ group}-\text{Average swelling degree of }\text{treatment group}\right)/\text{Average swelling degree }\text{of mice in the }\mathrm{Mod}\text{ group}\right]\times 100\%$$

#### 2.7.2 Acetic acid-induced capillary permeability

The mice were administered varying concentrations of the drug solutions intragastrically at a fixed time each morning, with a dose of 0.1 mL/10 g for seven consecutive days. The mice in the Mod group received distilled water, whereas those in the other groups were administered the appropriate doses of the test solutions. Then, 0.1 mL/10 g of 0.5% Evans blue saline solution was injected into the tail vein 1 h after the final administration, and 0.2 mL of 0.6% acetic acid solution was injected intraperitoneally. The mice were sacrificed after 20 min, and 6 mL of saline was injected intraperitoneally. The abdominal skin and muscles were massaged and then dissected, and the wash solution was collected. The optical density (OD) of the solution was measured at 590 nm. The inhibition rate (%) of the mouse exudate volume was calculated as follows (Akkol et al., 2022; Tian et al., 2021):

$$\text{Inhibition rate }\left(\%\right)=\left[\left(\text{Average OD value of the mice in }\mathrm{Mod}\text{ group}-\text{Average OD value of mice in the treated }\text{group}\right)/\text{Average OD value of mice in the }\mathrm{Mod}\text{ group}\right]\times 100\%$$

### 2.8 Analgesic activity

#### 2.8.1 Acetic acid-induced writhing

The mice were administered different concentrations of the drug solutions intragastrically at a fixed time each morning, with a dose of 0.1 mL/10 g for seven consecutive days. The mice in the Mod group received distilled water, whereas those in the other groups were given the appropriate doses of the sample solutions. Next, 0.1 mL/10 g of 0.6% acetic acid was injected intraperitoneally into each mouse 1 h after the final administration. The mice were then observed for writhing behavior, including hind limb and trunk extension, abdominal contraction, and high-hip posture. The number of twists exhibited by each mouse within 15 min was recorded, and the inhibition rate (%) was calculated as follows (Alam et al., 2020; Ukwubile et al., 2023):

$$\text{Inhibition Rate }\left(\%\right)=\left[\left(\text{Average number of twists in the }\mathrm{Mod}\text{ group}-\text{Average number of twists in }\text{the treated group}\right)/\text{Average number of }\text{twists in the }\mathrm{Mod}\text{ group}\right]\times 100\%$$

#### 2.8.2 Hot plate test

The mice were initially screened to determine their baseline pain threshold. Each mouse was placed on an intelligent hot plate apparatus set to 55 °C ± 0.5 °C. The mice with a baseline pain threshold ranging from 5 to 30 s were selected for the study. Following this, the mice were administered varying concentrations of the drug solutions intragastrically at a fixed time each morning, with a dose of 0.1 mL/10 g for each group. The mice in the Mod group received distilled water *via* gavage, whereas those in the other groups were administered the appropriate doses of the test solutions. The pain threshold of each mouse was measured at 30, 60, 90, and 120 min after the last dose. If a mouse exhibited no reaction, such as licking its hind paw, within 60 s, the pain threshold was recorded as 60 s. The results were then compared across the groups (Cui et al., 2022).

### 2.9 Grouping of animals with acute pharyngitis and drug administration

An acute pharyngitis rat model was established following the methods described in previous studies (Hu et al., 2022; Miao et al., 2018; Xu et al., 2020). Briefly, a 10% aqueous ammonia solution was sprayed into the throat once daily at 9:00 a.m., with approximately 0.1 mL per session, for three consecutive days. A total of 108 rats (54 females and 54 males) were randomly assigned to nine groups (*n* = 12): Con group, Mod group, A group, AS group, MC group, KHJ-L group, KHJ-M group, KHJ-H group, and positive control amoxicillin (AC) group. The rats in the Con group received water spray at the same site as the rats in the Mod group and were treated with an equivalent volume of water. Based on the doses used in the anti-inflammatory and analgesic experiments in mice, the dose for KHJ-H was determined to be 22 g/kg. According to the equivalent dose ratio of 20 g for mice to 200 g for rats (7.0), the KHJ-H dose for the in the acute pharyngitis rat model was set at 15.4 g/kg. The doses for the A, AS, and MC groups were calculated to be 5.5, 11, and 4.4 g/kg, respectively. AC was administered to rats in the positive control group at a dose of 0.36 g/kg. The drug was administered once daily by intragastric gavage for four consecutive days. The rats were anesthetized 30 min after the final administration. Then, the blood was collected *via* the abdominal aorta, and then the pharyngeal tissues were excised.

#### 2.9.1 Histological examination

The histopathological changes in the pharyngeal tissue of each rat were assessed using the hematoxylin and eosin (H&E) staining method.

#### 2.9.2 Analysis of serum biochemical indexes

The serum IL-1β, IL-6, IL-10, and PGE2 levels were measured using respective commercial ELISA kits.

#### 2.9.3 Western blot analysis of pharyngeal tissues

The protein expression levels in the pharyngeal tissue of each rat were determined using Western blot analysis. Detailed dilutions of all primary antibodies, including COX-2, NF-κB p65, p-NF-κB p65, and β-actin, were used.

### 2.10 Molecular docking studies

We performed a molecular analysis of the core proteins in KHJ to further elucidate the molecular interactions between the major quantitative chemical constituents of bergenin, matrine, oxymatrine, and macaine in KHJ and the core genes COX-2, NF-κB p65, and p-NF-κB p65 in acute pharyngitis. The protein structures of the core targets were downloaded from the PDB database (https://www.rcsb.org/). The core proteins were subjected to deprotonation and ligand removal using PyMOL 2.4.0 software. The structures of the core components were retrieved using the PubChem (https://pubchem.ncbi.nlm.nih.gov/) database. The SDF files were downloaded and converted into PDB files using Open Babel 3.1.1. The protein receptors and small-molecule ligands were routinely processed using AutoDock 4.2.6 software and saved in PDBQT format. The molecular docking and binding energy calculations were carried out using the AutoDock Vina 1.1.2 run script. Some of the results were visualized using PyMOL and Discovery Studio software.

### 2.11 Statistical analysis

Statistical analysis was performed using SPSS 26.0 (International Business Machines Corporation), and the results were expressed as mean ± standard deviation (SD). The data meeting the criteria for normality and homogeneity of variance were analyzed using one-way analysis of variance. Data not meeting these criteria were analyzed using non-parametric tests. Statistical significance was set at *P* < 0.05. Graphs were created using GraphPad Prism 9.5 software (GraphPad Software Corporation).

## 3 Results

### 3.1 Validation of the fingerprint evaluation method

The precision, repeatability, and stability of the fingerprint evaluation method were validated. For precision testing, six consecutive injections of the KHJ solution were analyzed, using peak 11 used as a reference. The relative standard deviation (RSD) values for relative retention times (RRTs) and relative peak areas (RPAs) of 22 common fingerprint peaks were did not exceed 0.64% and 2.81%, respectively. For repeatability, six KHJ solutions were injected. The results showed that RRTs and RPAs did not exceed 0.18% and 2.74%, respectively. The stability testing of the KHJ solution stored at 4 °C was conducted at intervals of 0, 2, 4, 6, 8, 12, and 24 h, with RRTs and RPAs remaining within 0.62% and 2.95%, respectively. These results indicated that the fingerprint evaluation method was reliable (Supplementary Table S2).

### 3.2 Establishment of fingerprint chromatograms for KHJ

**FIGURE 2:**
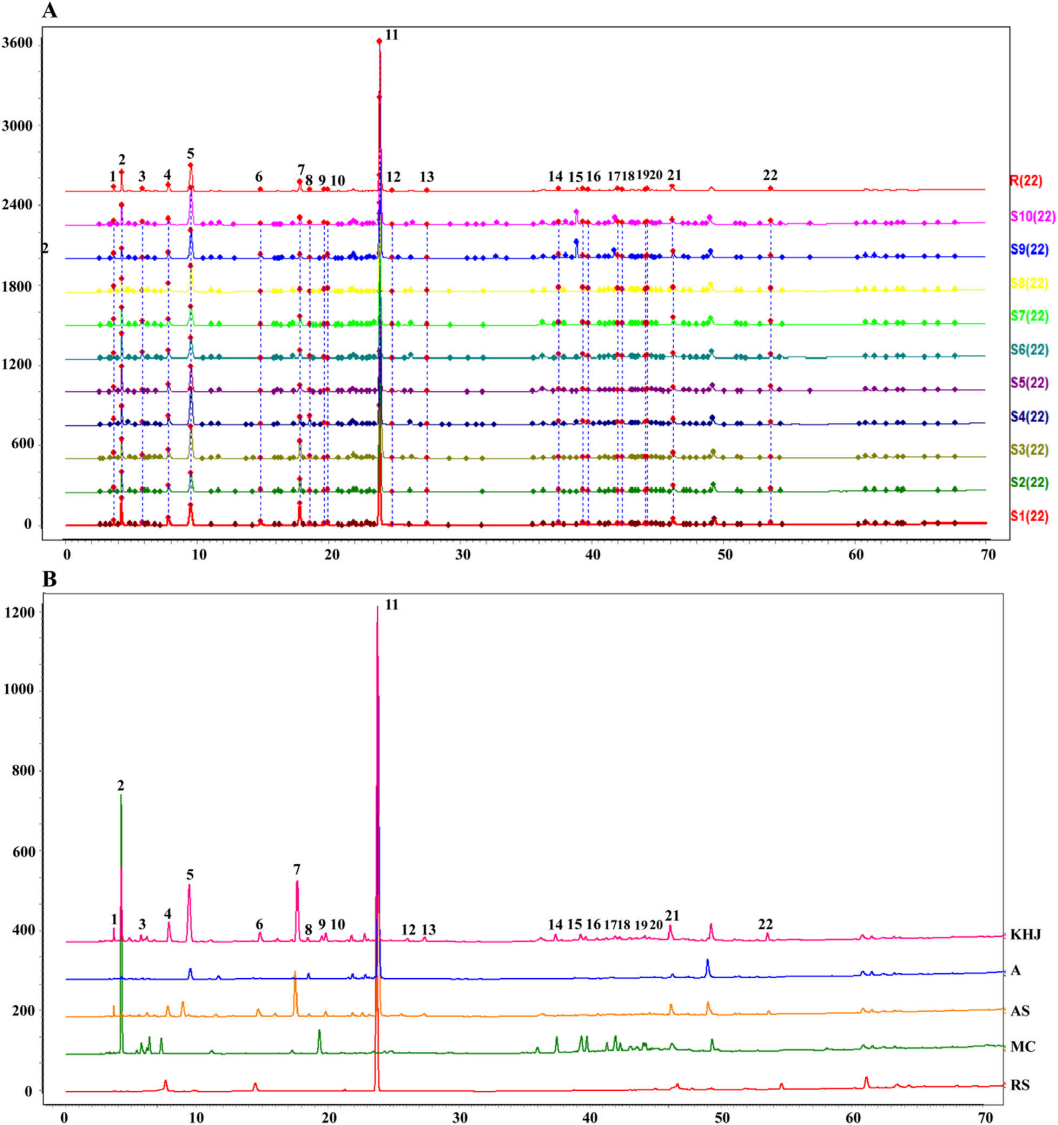
**(A)** 10 batches of fingerprint spectra of KHJ; **(B)** HPLC chromatogram of KHJ, A, AS, MC, RS (Reference Substance); 4: Matrine; 6: Oxymatrine; 11: Bergenin; 22: Macaine.

The fingerprints were established for KHJ by preparing test solutions from ten batches each of A, AS, MC, and the complete KHJ formula (Figure 2; Supplementary Figures S1–S3). The samples were analyzed under specified chromatographic conditions, and the chromatograms were recorded. Using the Chinese Medicine Chromatographic Fingerprint Similarity Evaluation Software (2012 Edition), the sample chromatograms (A1, AS1, MC1, and S1) served as references to generate control fingerprint chromatograms (R) by the median method. A total of 22 common peaks were identified in the HPLC fingerprint of the 10 KHJ batches. The comparisons with HPLC fingerprint profiles of mixed reference samples identified peak 4 as matrine, peak 6 as oxymatrine, peak 11 as bergenin, and peak 22 as macaine. Of the 22 peaks identified, 13 were (1, 3–8, 10–13, and 21–22) attributed to AS, 4 (3, 5, 8, and 11) to S alone, and 8 (9 and 14–20) to MC. The results demonstrated that the fingerprint spectrum remained consistent before and after compatibility, with no new components showing significantly higher response values. Similarity evaluation results were shown in Supplementary Table S3.

### 3.3 Determination of chemical composition

#### 3.3.1 Method validation

The method was validated as outlined in Section 2.4. The precision was assessed by analyzing six parallel samples of the reference solution, with RSD values for the peak areas of the four compounds ranging from 0.27% to 0.74%. The repeatability was evaluated by injecting six parallel samples of KHJ. The RSD values for the contents of the four compounds ranged from 1.24% to 2.44%. The stability testing of the KHJ solution stored at 4 °C was performed after 0, 2, 4, 6, 8, 12, and 24 h. The sample remained stable for 24 h, with RSD values for the peak areas of the four compounds not exceeding 1.92%. The recovery was determined using the standard addition method with six parallel samples at each concentration, corresponding to 1.0 times the concentration of the mixed reference solution B. The recoveries of the four compounds ranged from 97.43% to 102.76%, with RSD values of 0.59%–2.24%. Standard curves were constructed by plotting 2, 5, 10, 15, and 20 µL of the mixed reference solution by taking the reference sample injection volume as the horizontal axis (*X*) and the peak area as the vertical axis (*Y*). Each component exhibited good linearity within its respective range. The calibration curves for all four compounds demonstrated excellent linearity, with correlation coefficients (*r*) ranging from 0.9996 to 1.0000 (Supplementary Table S3).

#### 3.3.2 Changes in the content of indicator components before and after compatibility of KHJ

The content of four key compounds was analyzed in ten batches each of KHJ, A, and AS using the procedure described in Section 2.4. The results (Table 1) showed that the content of bergenin and matrine remained nearly constant after compatibility of KHJ. In contrast, the content of oxymatrine decreased, and the content of matrine increased. However, the total content of matrine and oxymatrine remained relatively unchanged, suggesting that the dissolution of these indicator components was mutually affected by their compatibility. The detailed results are presented in Table 2.

**TABLE 1:** Determination results of each component content (mg/g).

Sample	Matrine	Oxymatrine	Bergenin	Macaine
A1	—	—	5.9677	—
A2	—	—	11.9257	—
A3	—	—	11.0286	—
A4	—	—	9.9596	—
A5	—	—	16.6185	—
A6	—	—	10.6611	—
A7	—	—	8.2020	—
A8	—	—	10.6743	—
A9	—	—	10.6875	—
A10	—	—	7.2112	—
AS1	4.3672	2.6041	6.6287	0.2218
AS2	5.8293	3.3698	8.4705	0.3175
AS3	4.2709	1.5443	9.6965	0.1040
AS4	4.9417	1.6203	11.8171	0.1707
AS5	6.1923	1.9574	16.0757	0.5895
AS6	4.6399	0.6430	10.4237	0.2068
AS7	3.9496	0.9824	8.7787	0.4877
AS8	5.9961	1.5224	10.6755	0.3254
AS9	5.6773	3.6433	12.3010	0.2581
AS10	4.3585	8.6659	11.5042	0.7289
KHJ1	5.9359	2.2527	8.0001	0.3437
KHJ2	6.7432	1.8887	12.0146	0.3576
KHJ3	6.5199	0.4067	9.6929	0.1246
KHJ4	5.9661	0.5600	8.9143	0.2161
KHJ5	7.3757	0.8103	14.8421	0.6663
KHJ6	7.3473	0.3941	10.1062	0.4235
KHJ7	5.3573	0.337	7.5065	0.3571
KHJ8	6.7473	0.452	7.7300	0.3023
KHJ9	6.0874	3.1372	14.1486	0.3048
KHJ10	3.4168	0.5458	8.6634	0.3263

**TABLE 2:** Changes in the content of indicator components of the KHJ (mg/g).

Sample	Matrine	Oxymatrine	Bergenin	Macaine
A	—	—	10.2936 ± 2.7630	—
AS	5.0223 ± 0.7833	2.6553 ± 2.2032	10.6372 ± 2.4421	0.3410 ± 0.1894
KHJ	6.1497 ± 1.0938	1.0784 ± 0.9359	10.1619 ± 2.5079	0.3422 ± 0.1336

Results are mean ± SD (n = 10).

### 3.4 Anti-inflammatory and analgesic activity before and after compatibility of KHJ

#### 3.4.1 Xylene-induced ear edema

**FIGURE 3:**
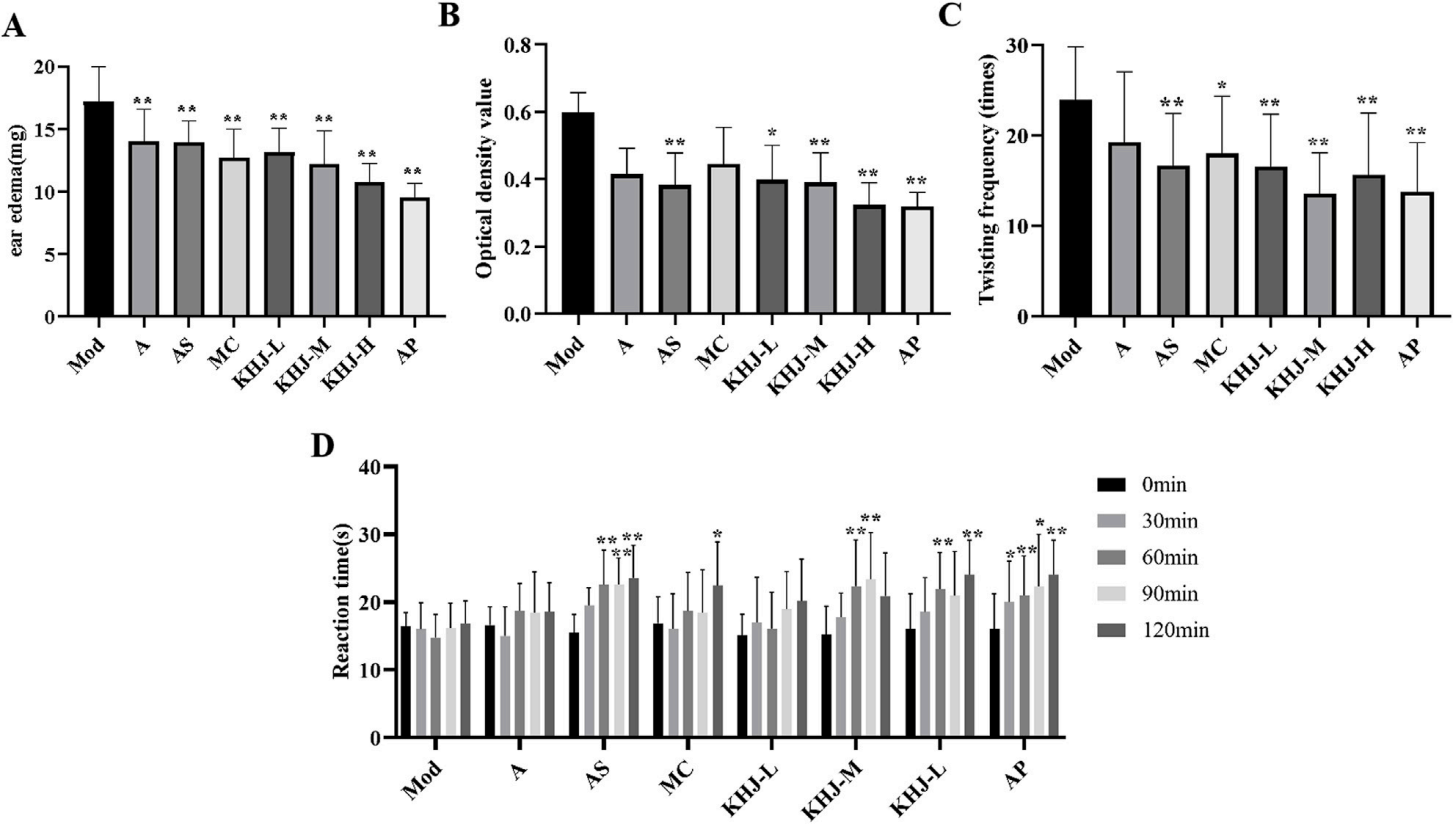
Anti-inflammatory and analgesic activity before and after the compatibility of the KHJ. **(A)** Xylene-induced ear oedema; **(B)** Optical density value; **(C)** Number of acetic acid induced writhing; **(D)** Reaction time in hot-plate test; Results are mean ± SD (n = 12), *P < 0.05, **P < 0.01 vs. Model.

The results from the xylene induced ear edema test are illustrated in Figure 3A. The A, AS, MC, KHJ-H, KHJ-M, and KHJ-L groups all exhibited significant inhibition of ear edema compared with the Mod group (*P* < 0.01). Among these, the KHJ-H group demonstrated the most pronounced effect. The auricular inhibition rates (%) were ranked as follows: KHJ-H group, KHJ-M group, MC group, KHJ-L group, AS group, and A group.

#### 3.4.2 Acetic acid-induced capillary permeability

Figure 3B shows the impact of acetic acid on mouse peritoneal capillary permeability in mice. Treatment with AS and varying doses of KHJ significantly reduced peritoneal vascular permeability compared with that in the Mod group (*P* < 0.05, *P* < 0.01). The A and MC groups displayed some inhibitory effects on vascular permeability, but the differences were not statistically significant (*P* > 0.05). The inhibition rates (%) from the highest to the lowest were as follows: KHJ-H group, AS group, KHJ-M group, KHJ-L group, A group, and MC group.

#### 3.4.3 Acetic acid–induced writhing test

As shown in Figure 3C, the AS, MC, and KHJ treated groups exhibited significant reductions in the twisting response of mice compared with the Mod group (*P* < 0.05, *P* < 0.01). The A group demonstrated some inhibitory effect, but it was not statistically significant (*P* > 0.05). The twist inhibition rates (%) were ranked from the highest to the lowest as follows: KHJ-M group, KHJ-H group, KHJ-L group, AS group, MC group, and A group.

#### 3.4.4 Determination of the antinociceptive activity using the hot plate test

Figure 3D illustrates the antinociceptive activity assessed using the hot plate test. The pain thresholds of mice in the AS group were significantly prolonged at 60, 90, and 120 min after administration (*P* < 0.01). The MC group showed a significant increase in pain thresholds after 120 min (*P* < 0.05). The KHJ-M and KHJ-H groups exhibited prolonged pain thresholds after 60 and 90 min, and 60 and 120 min, respectively. These results indicated that the AS, MC, KHJ-M, and KHJ-H groups demonstrated significant inhibitory effects on nociceptive responses.

### 3.5 Effect of KHJ on acute pharyngitis before and after compatibility

#### 3.5.1 Behavioral status and appearance index score of rats

**FIGURE 4:**
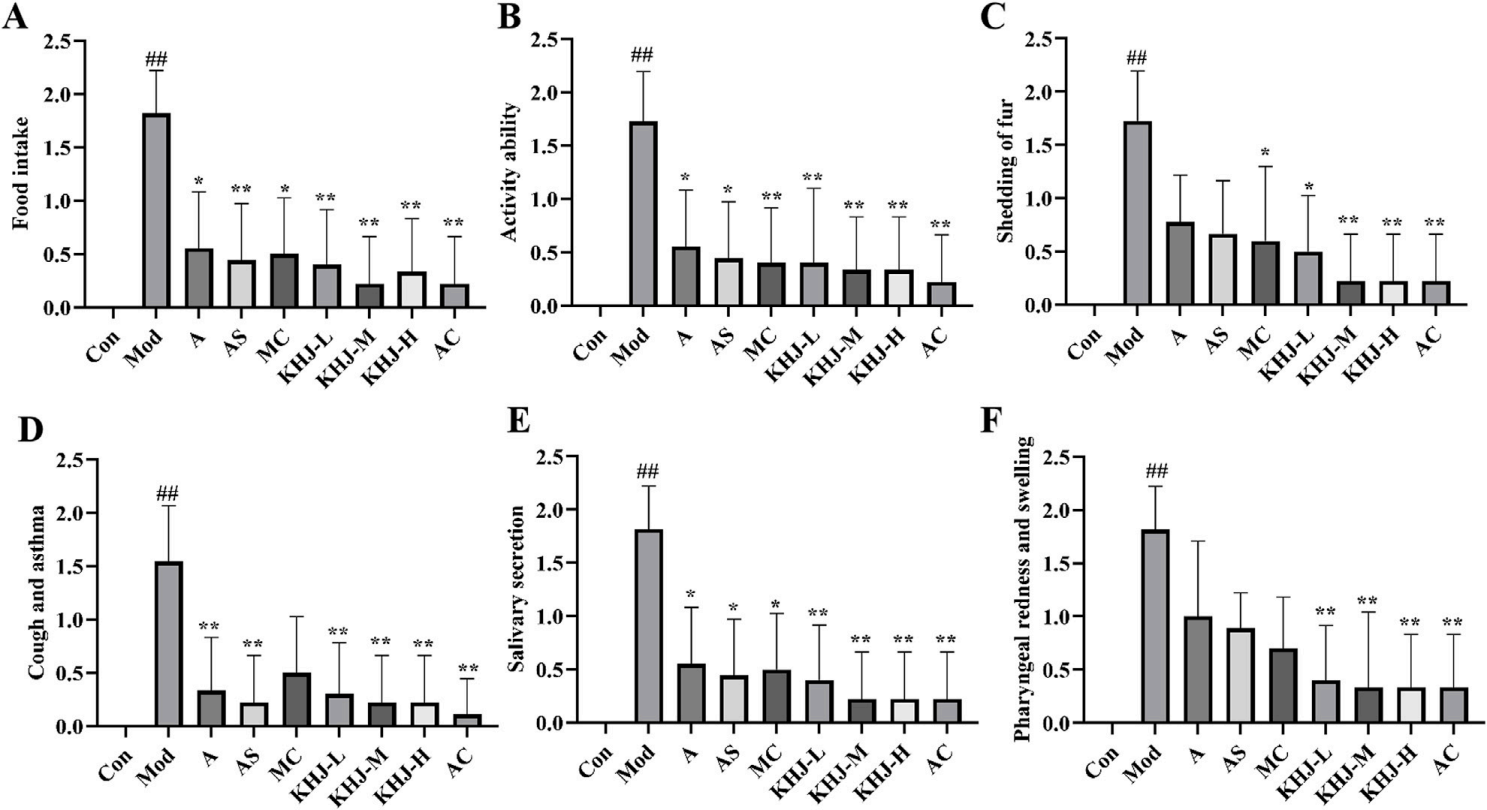
Rat behavioral status and apparent index score. **(A)** Food intake; **(B)** Activity ability; **(C)** Mouth fur shedding; **(D)** Cough and asthma; **(E)** Salivary secretion; **(F)** Pharyngeal redness and swelling. Results are mean ± SD (n = 12)*,* ^*#*^ *P <* 0.05,^*##*^ *P <* 0.01 vs. Control, *P < 0.05, **p < 0.01 vs. Model.

The apparent index scores for acute pharyngitis were assessed on the seventh day of the experiment according to the criteria outlined in the “Guidelines for the Preparation of Acute Pharyngitis Animal Models” (Cui et al., 2022) and based on the scoring criteria provided in Table 3. The observations included feeding behavior, activity levels, muzzle fur shedding, coughing and wheezing, salivation, and the area of pharyngeal swelling. The changes in these indicators after administration were compared with those in the model group to evaluate the therapeutic effects of the complete KHJ formula and its individual components on acute pharyngitis. The Mod group exhibited significantly higher scores across several behavioral and appearance indices compared with the Con group (*P* < 0.01). The rats in the Mod group exhibited decreased food intake, reduced activity, increased scratching and shedding of muzzle fur, as well as coughing, increased salivation, and pharyngeal redness and swelling. These observations confirmed the successful establishment of acute pharyngitis in the Mod group *via* ammonia water stimulation. The scores for each treatment group were lower than those for the Mod group, indicating that both the complete formula and its individual components improved the clinical symptoms of acute pharyngitis. KHJ-H, KHJ-M, and KHJ-L demonstrated significant therapeutic effects in alleviating the symptoms of acute pharyngitis (*P* < 0.05, *P* < 0.01) (Figure 4).

**TABLE 3:** Scoring criteria for behavioural status and epigenetic indicators in rats.

Symptom score	Food intake	Activity ability	Mouth fur shedding	Cough and asthma	Salivary secretion	Pharyngeal redness and swelling
0	Normal	Normal	No	No	No	Normal
1	Mild	Mild	Slight	Mild	Minor	Mild
2	Significant	Significant	Obvious	Clear	Excessive	Severe

#### 3.5.2 Pathology of rat pharyngeal tissues

**FIGURE 5:**
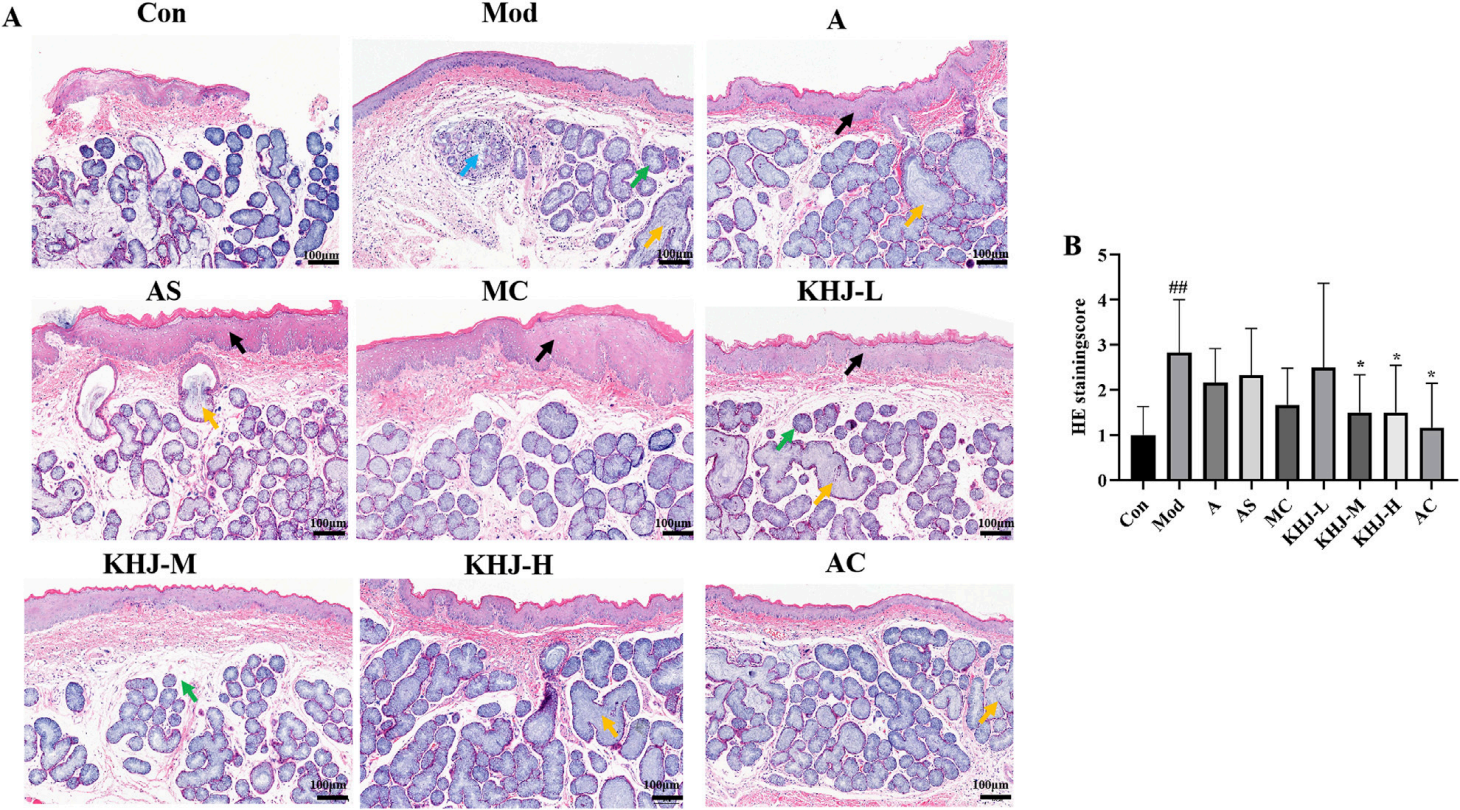
**(A)** HE staining of rat pharyngeal tissue (×100); **(B)** HE staining score of rat pharyngeal tissue, results are mean ± SD (n = 6),^*##*^ *P <* 0.01 vs. Control, *P < 0.05, vs. Model. Black arrow: mucosal epithelial thickening; yellow arrow: mucous gland hypertrophy; green arrow: mdema of lamina propria; blue arrow: mucous gland degeneration and necrosis.

The effects of KHJ, before and after compatibility, on acute pharyngitis in rats were evaluated using H&E staining. Light microscopic observations revealed significant edema in the submucosa, hypertrophy and necrosis of glandular follicular cells, inflammatory cell infiltration, and vascular dilation and congestion in the pharyngeal tissues of rats in the Mod group compared with the Con group. The pharyngeal tissues in the other treatment groups exhibited varying degrees of improvement compared with those in the Mod group. The KHJ-L group displayed less pronounced recovery, characterized by persistent mucosal epithelial hyperplasia, enlargement of the mucosal glands, and mucosal lamina propria edema. The A and AS groups demonstrated a moderate recovery trend, with occasional mucosal epithelial hyperplasia or enlargement of the mucosal glands. The MC, KHJ-M, and KHJ-H groups exhibited marked improvement with significant reductions in pathological changes. Representative light microscopy images of the pharyngeal tissues from each group under light microscopy are shown in Figure 5A.

The H&E staining scores for the pharyngeal tissues were based on four factors: mucosal epithelial hyperplasia, mucosal lamina propria edema, inflammatory cell infiltration, and mucosal gland hypertrophy. The Mod group exhibited significantly higher HE scores compared with the Con group, indicating successful model establishment. Each drug administration group had reduced HE scores compared with the Mod group. The HE scores for the KHJ-H and KHJ-M groups were significantly lower than those for the Mod group (*P* < 0.05), reflecting substantial improvement in the pharyngeal tissues. These findings indicate that while acute pharyngitis symptoms were evident after ammonia water stimulation,the drug interventions led to the recovery of symptoms in the pharyngeal tissues (Figure 5B).

#### 3.5.3 Serum IL-1β, IL-6, IL-10, and PGE2 levels in rats

**FIGURE 6:**
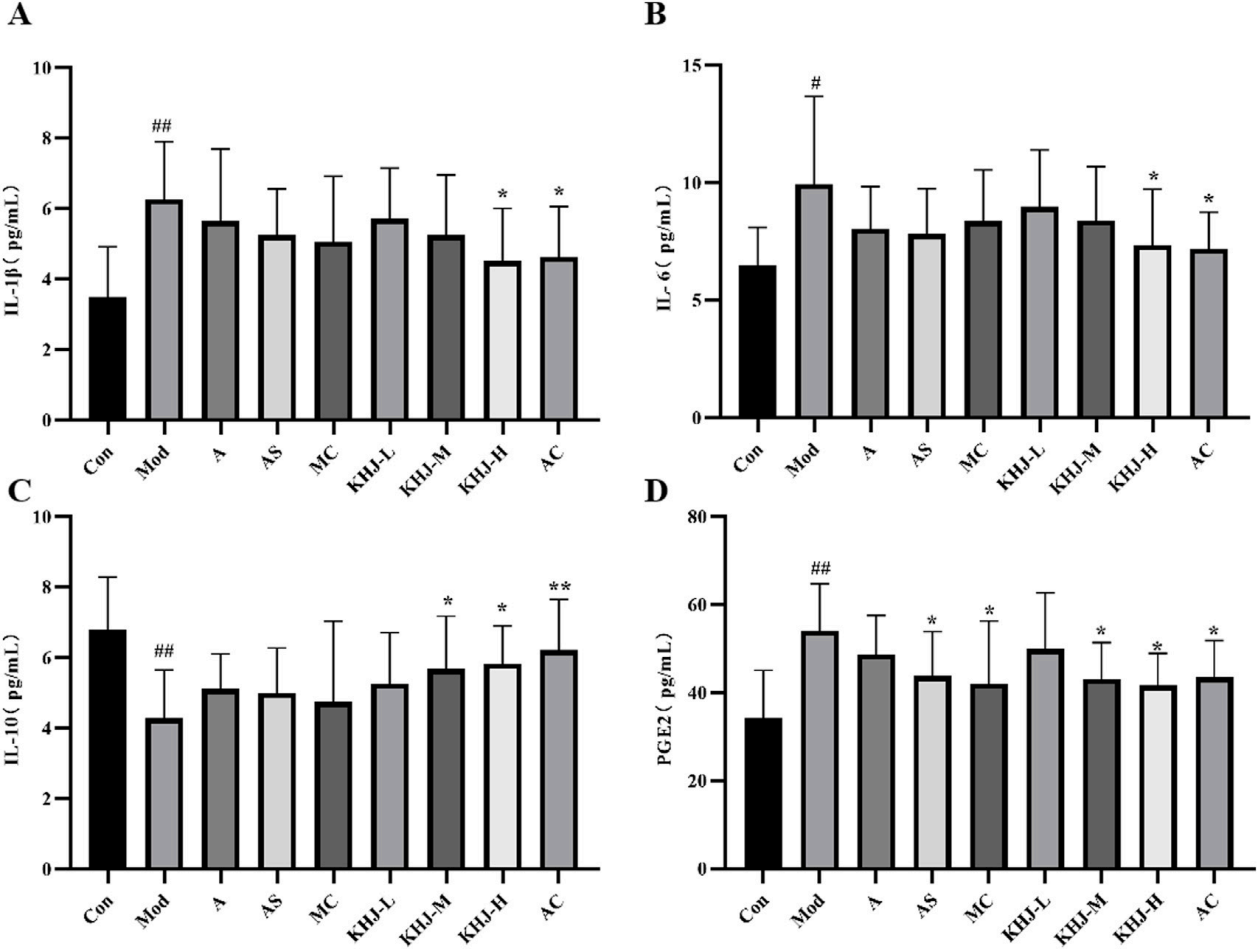
KHJ before and after compatibility on inflammatory cytokines in serum of acute pharyngitis model rats. **(A)** The content of IL-1β; **(B)** The content of IL-6; **(C)** The content of IL-10; **(D)** The content of PGE2. Results are mean ± SD (n = 12), ^*##*^ *P* < 0.01 vs. Control, *P < 0.05, **P < 0.01 vs. Model.

The levels of inflammatory cytokine levels, including IL-1β, IL-6, IL-10, and PGE2, in rat serum were measured using an ELISA kit. As shown in Figure 6, the Mod group exhibited significantly higher IL-1β, IL-6, and PGE2 levels than the Con group (*P <* 0.05, *P <* 0.01) and significantly lower IL-10 levels (*P <* 0.01). This indicated that ammonia water stimulation led to the increased release of inflammatory factors in rats.

Each treatment group demonstrated reduced IL-1β, IL-6, and PGE2 levels, along with increased IL-10 levels, compared with the Mod group. This suggested that the treatments were effective in reducing inflammation. Specifically, the AS group showed a significant reduction in PGE2 levels (*P <* 0.05), as did the MC group (*P <* 0.05). The KHJ-M group demonstrated increased IL-10 levels (p < 0.05) and significantly decreased PGE2 levels (*P <* 0.05). Similarly, the KHJ-H group exhibited significant increases in IL-10 levels (*P* < 0.05) and significant decreases in IL-1β, IL-6, and PGE2 levels (*P* < 0.05).

#### 3.5.4 Expression of COX-2, NF-κB p65, and p-NF-κB p65 proteins in rat pharyngeal tissues

**FIGURE 7:**
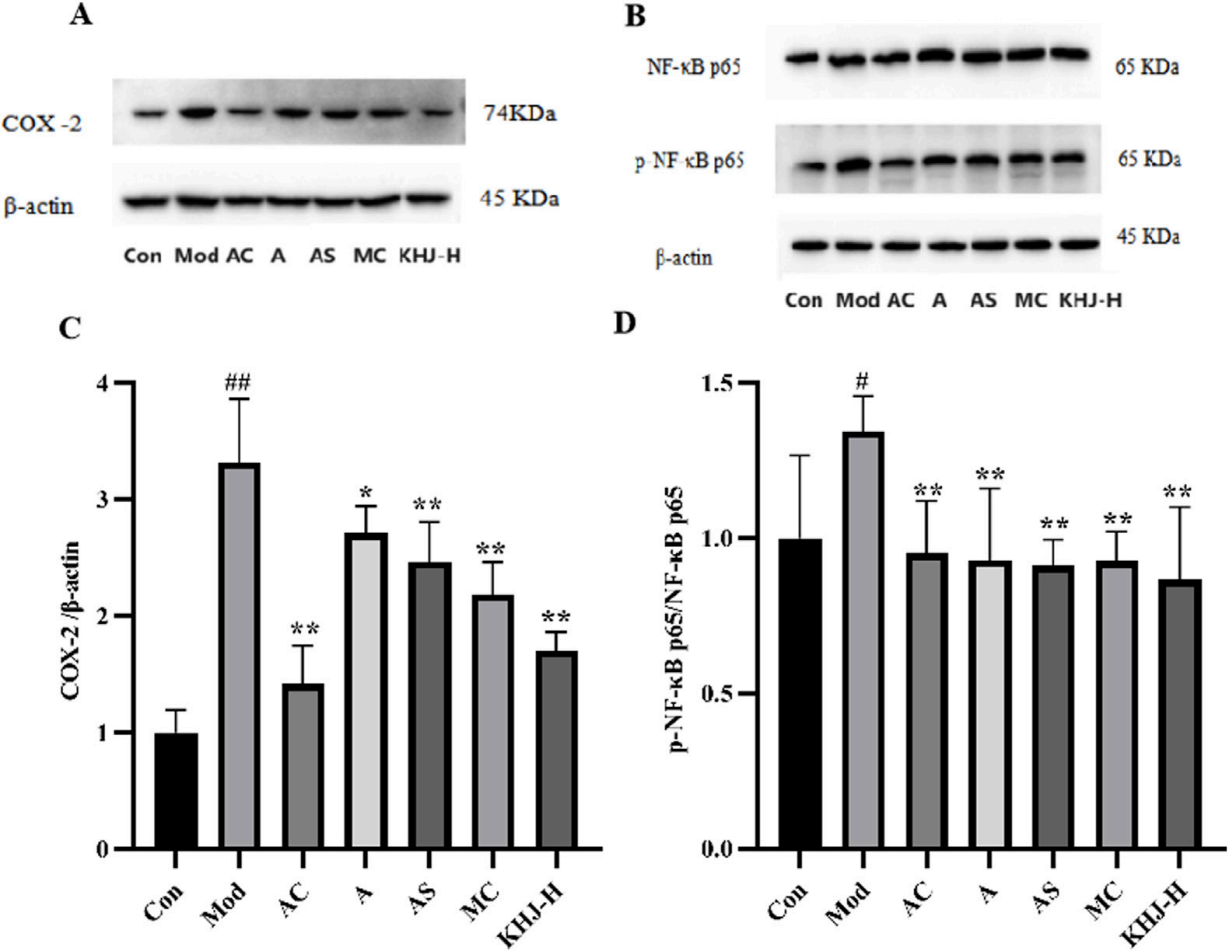
The effect of COX-2, p65 and p-p65 protein expression in rat pharyngeal tissues. **(A)** Representative bands of COX-2 and β-actin; **(B)** Representative bands of NF-κB p65, p-NF-κB p65, and β-actin; **(C)** The relative protein expression of COX-2/β-actin; **(D)** The relative protein expression of p-NF-κB p65/NF-κB p65. ^*#*^ *P* < 0.05*,* ^*##*^ *P* < 0.01 vs. Control, *P < 0.05, **P < 0.01 vs. Model.

The protein expression levels of COX-2 and the ratio of p-NF-κB p65 to NF-κB p65 in the pharyngeal tissues were assessed. The Mod group exhibited significantly higher COX-2 and p-NF-κB p65/NF-κB p65 protein levels compared with the Con group (*P* < 0.01, *P* < 0.05). A, AS, MC, and KHJ-H treatments resulted in significant downregulation of COX-2 and p-NF-κB p65/NF-κB p65 protein levels (*P* < 0.05, *P* < 0.01) compared with those in the Mod group (Figure 7). These results suggested that both the complete KHJ formula and its individual components effectively inhibited the overexpression of inflammatory signaling pathways and COX-2 and NF-κB p65/NF-κB p65 proteins stimulated by inflammation. This implied that KHJ, in both its formula and individual components, might aid in the treatment of acute pharyngitis by modulating the COX-2/NF-κB signaling pathway.

#### 3.5.5 Molecular docking results

At the molecular level, the active components of KHJ exerted therapeutic effects against acute pharyngitis by modulating the levels of key inflammatory mediators, including COX-2, NF-κB p65, and p-NF-κB p65, *via* a multi-target mechanism. In this study, four components were quantitatively analyzed and their interactions with three critical genes associated with acute pharyngitis were evaluated using molecular docking techniques. This process yielded a total of 12 docking configurations. The results demonstrated that matrine exhibited strong binding affinity for COX-2 (−7.99 kcal/mol). Oxymatrine also displayed high binding affinity for COX-2 (−8.50 kcal/mol). This study demonstrated that bergenin exhibited moderate binding affinity for COX-2 (−7.60 kcal/mol). However, macaine exhibited the highest binding affinity for COX-2 (−8.80 kcal/mol). On the contrary, both bergenin and macaine exhibited reduced binding affinities for NF-κB p65. These findings indicate a robust interaction between the key bioactive compounds and their core molecular targets. The binding affinity data are presented in Table 4.

**TABLE 4:** Docking binding energy of four quantitative chemical components to three targets.

Compound	Binding affinity (K cal/mol)		
	NF-κB p65	p-NF-κB p65	COX-2
Matrine	−6.10	−7.98	−7.99
Oxymatrine	−6.11	−8.08	−8.50
Bergenin	−3.17	−7.45	−7.60
Macaine	−4.78	−7.38	−8.80

**FIGURE 8:**
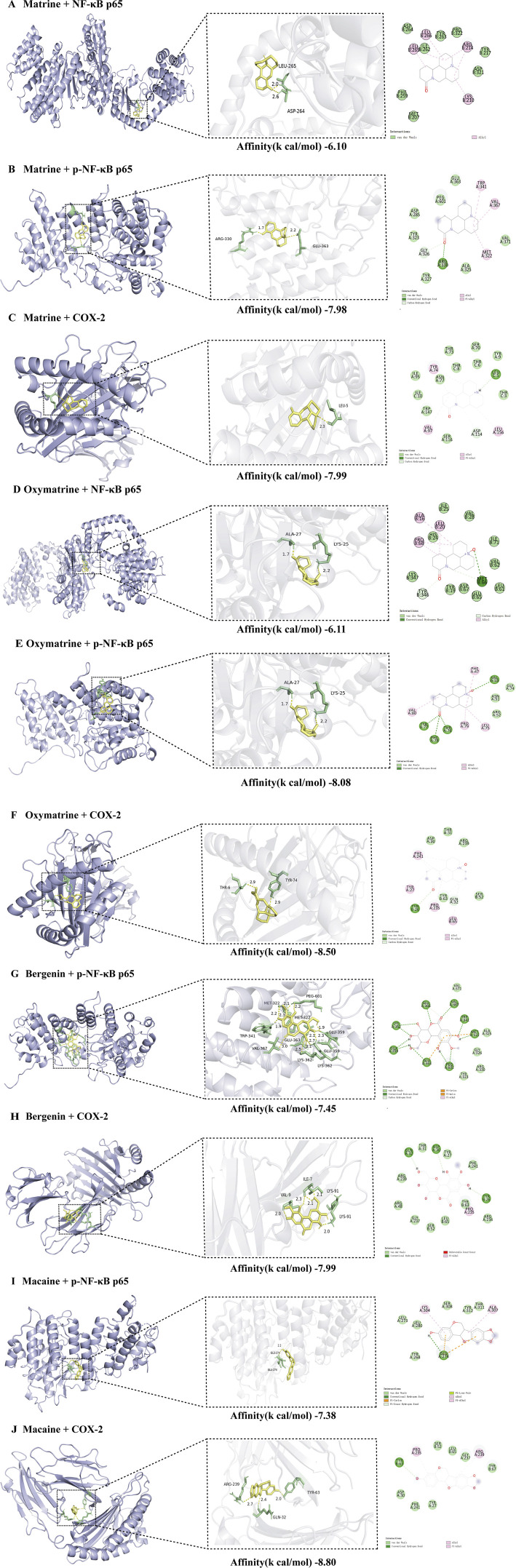
**(A)** Molecular docking of Matrine to NF-κB p65 and its 2D chemical structure; **(B)** Molecular docking of Matrine to p-NF-κB p65 and its 2D chemical structure; **(C)** Molecular docking of Matrine to COX-2 and its 2D chemical structure; **(D)** Molecular docking of Oxymatrine to NF-κB p65 and its 2D chemical structure; **(E)** Molecular docking of Oxymatrine to p-NF-κB p65 and its 2D chemical structure; **(F)** Molecular docking of Oxymatrine to COX-2 and its 2D chemical structure; **(G)** Molecular docking of Bergenin to p-NF-κB p65 and its 2D chemical structure; **(H)** Molecular docking of Bergenin to COX-2 and its 2D chemical structure; **(I)** Molecular docking of Macaine to p-NF-κB p65 and its 2D chemical structure; **(J)** Molecular docking of Macaine to COX-2 and its 2D chemical structure.

Molecular docking visualizations were performed for the target proteins to further elucidate the aforementioned interactions. The results demonstrated that the active components of KHJ achieved stable binding through multiple interactions with key residues of the target proteins (Figures 8A-J). The docking scores indicated that matrine, oxymatrine, bergenin, and macaine exhibited high binding affinity with COX-2.

Matrine primarily interacted with residues THR73, ASP114, SER116, SER70, ILE95, TRP147, and TYR9 of the A-chain, as well as THR3/6/8 and ILE10 of the C-chain, within the active pocket of COX-2, resulting in multiple van der Waals interactions. LEU5 formed conventional hydrogen bonds with the polar groups of matrine, thereby significantly enhancing stability. Meanwhile, TYR74, VAL97, and LEU156 interacted with the hydrophobic backbone of matrine through alkyl and Pi-alkyl interactions, demonstrating strong spatial complementarity (Figure 8C).

Similarly, oxymatrine displayed stable interactions with the key functional domains of COX-2 through diverse noncovalent bonds. It exhibited conventional hydrogen bond interactions with TYR26, hydrophobic contacts with PHE47, VAL80, PRO79, and LEU75, and multiple van der Waals interactions with ASP30, THR31, ARG239, TYR63, and SER52 (Figure 8F).

Bergenin formed conventional hydrogen bonds with ASP30, TYR26, and GLN32 of COX-2, Pi-alkyl interactions with PRO235, and van der Waals interactions with ARG239/48/234, THR31, TYR27/63, GLY237, SER52, LEU65, and PHE241 (Figure 8H).

Macaine exhibited conventional hydrogen bonding with TYR63, alkyl and Pi-alkyl interactions with PRO235 and ARG239, respectively, and van der Waals interactions with ASP30, THR31, TYR27/67, and SER52, among other relevant residues. As illustrated in Figures 8I,J, the key residues such as LEU65 and GLY237 were located within the specified binding regions, further confirming the precise molecular recognition between KHJ components and the COX-2 active site.

## 4 Discussion

This study established HPLC fingerprinting and quantification methods for analyzing the main components of both the complete KHJ formula and its individual components. The fingerprint analysis revealed no significant changes in the number of chromatographic peaks before and after compatibility. However, the interactions between indicator components were observed, with the compatibility process resulting in an increase in matrine content and a decrease in oxymatrine content. Despite these changes, the total content of these components remained relatively stable, suggesting a possible conversion between them. The animal studies demonstrated that the complete KHJ formula exhibited superior anti-inflammatory and analgesic effects compared with its individual components. Additionally, the complete formula was more effective in treating acute pharyngitis, thus significantly reducing inflammatory responses in rats. This efficacy was attributed to the modulation of the NF-κB/COX-2 signaling pathway. These findings validate the use of KHJ as a combined formulation and provide insight into its mechanism of action in treating acute pharyngitis.

The fingerprint profile experiment revealed consistent chromatographic peak patterns before and after the compatibility of KHJ formula. The assay experiments indicated that the compatibility process led to an increase in matrine content and a decrease in oxymatrine content, suggesting interactions between the components in the KHJ formula. Although the sum of the contents remained unchanged, this interaction implied a potential conversion between the two components. Oxymatrine, a significant alkaloid found in S, possesses anti-inflammatory and analgesic properties but is also associated with liver damage, nerve damage, and gastrointestinal reactions (Yu et al., 2022). The toxicity of S is known to increase with longer cooking durations (Chen et al., 2017). A dose-response relationship between efficacy and toxicity for alkaloids. Based on these experimental results, it can be inferred that the post-formulation compatibility may enhance efficacy while reducing the toxicity of S to some extent.

Xylene is an inflammatory agent that induces inflammation and swelling when applied to the mouse earlobe, leading to the release of inflammatory mediators (Righi et al., 2021). Acetic acid, acts as a pro-inflammatory factor at low concentrations, triggering acute inflammatory responses. Changes in capillary permeability can be assessed by measuring the permeability of mouse peritoneal fluid following the intravenous injection of Evans blue dye (Dönmez et al., 2020; He et al., 2019). Xylene and acetic acid are widely used to model earlobe swelling and increased abdominal capillary permeability, respectively, rendering making them classic tools for anti-inflammatory research (Zhang et al., 2024). These models offer high time efficiency and success rates, facilitating the effective evaluation of the effects of drugs on acute inflammation. The present study, we examined the anti-inflammatory effects of both the complete KHJ formula and its individual components of KHJ using these established anti-inflammatory models. All treatment groups demonstrated a significant reduction in ear swelling compared with the Mod group, with the complete formula group displaying the most pronounced effect. In the mouse abdominal capillary permeability test, while the A group used alone or in combination with MC did not exert significant anti-inflammatory effects. However, both AS and the complete KHJ formula significantly inhibited abdominal capillary permeability. Furthermore, the complete KHJ formula exhibited superior anti-inflammatory effects compared with AS, indicating a dose-response relationship.

Inflammation is closely associated with pain, and many inflammatory diseases often involve persistent pain responses. The hot plate test is used to evaluate the effects of drugs on central pain processing through measuring the changes in pain threshold by assessing the sensitivity to thermal stimulation (Sun et al., 2024; Hossain et al., 2017). The writhing test involves injecting acetic acid into the peritoneal cavity of the mouse, which irritates both the visceral and parietal layers, resulting in to pain. The number of writhing movements in mice reflects the intensity of this pain (Ariyo et al., 2020; Razafindrakoto et al., 2020). In the hot plate and writhing tests conducted in this study, the combined formulas of AS, MC, and KHJ exhibited a significant prolongation of pain threshold and reduction in writhing responses in mice. These findings suggested the substantial analgesic effect of KHJ. Specifically, the main drug A, when combined with S, enhanced the analgesic effect compared with A alone. Moreover, combining A with MC further improved the analgesic effect. These analgesic trials support the rationality of using KHJ, demonstrating not only its remarkable in anti-inflammatory and analgesic effects but also the advantage of combining the main medicinal composition AS with auxiliary components such as MC. This study confirmed the scientific basis for KHJ formulation and validated its combined use for enhanced therapeutic efficacy.

The present study confirmed the anti-inflammatory and analgesic activities of KHJ using classical pharmacological models, including xylene-induced mouse ear edema, acetic acid--induced capillary permeability, hot plate tests, and acetic acid-induced writhing responses. These models serve as general screening tools and do not directly replicate the complex etiology or localized pharyngeal pathology of acute pharyngitis. However, the core pathological processes evaluated in these models, namely increased vascular permeability, tissue edema, and inflammatory pain mediated by inflammatory mediators such as prostaglandins and histamine, are highly relevant to the key clinical manifestations of acute pharyngitis, including mucosal swelling, hyperemia, and pain (Watkins and Maier, 2023; Abdulkhaleq et al., 2018; Bathala and Eccles, 2013). Thus, the results demonstrate that KHJ possesses significant fundamental anti-inflammatory and analgesic properties of KHJ, thus providing an important pharmacological basis for its potential to alleviate the core symptoms of acute pharyngitis. Subsequent investigations further validated these effects in animal models that more closely mimic the pathophysiology of acute pharyngitis.

Acute pharyngitis is a common condition often triggered by bacterial, viral, or environmental factors (Zhu et al., 2021). TCM typically addresses acute pharyngitis by focusing on clearing heat, detoxifying, and promoting blood circulation to alleviate stasis (Wang et al., 2024). In clinical practice, KHJ has been demonstrated to effectively relieve the symptoms of acute pharyngitis, which confirms its effectiveness and safety (Pang et al., 2023). Animal models for acute pharyngitis are often established using methods such as ammonia spray or infection models (Lu et al., 2024). Ammonia stimulation triggers an inflammatory response characterized by the aggregation of inflammatory cells and the release of pro-inflammatory factors, with macrophages being the primary source of TNF-α, which in turn promotes the production of various cytokines (Ran et al., 2021). IL-6 activates mononuclear macrophages and impacts immune responses, whereas IL-1β overexpression causes tissue damage, edema, and exacerbate responses in rats with acute pharyngitis through interactions with TNF-α and IL-6 (Zhang et al., 2021). IL-10, an immunosuppressive factor, plays a crucial role in regulating systemic functions, whereas PGE2 impacts the temperature regulation center, stimulating peripheral nerve endings and thus leading to fever.

In the experimental studies assessing the pharmacological effects of KHJ on acute pharyngitis before and after compatibility, all drug groups exhibited significant reductions in serum IL-1β, IL-6, and PGE2 levels, besides a notable increase in IL-10 levels. The KHJ-H complete formula demonstrated the most pronounced effect. These results suggested that the combined use of main and adjuvant drugs in the KHJ complete formula led to a more robust therapeutic effect on rats with acute pharyngitis. This confirmed the rationale behind the compatibility of the KHJ formula, highlighting its efficacy in inhibiting the expression of pro-inflammatory factors and promoting the expression of anti-inflammatory factors to effectively regulate inflammatory responses.

The NF-κB inflammatory response family plays a crucial role in both innate and adaptive immunity, thus influencing the differentiation, proliferation, and survival of multicellular organisms. Specifically, NF-κB p65 is central to the inflammatory response (Mitchell et al., 2016). Upon activation, NF-κB p65 rapidly translocates to the nucleus, where it regulates the expression of inflammatory cytokines such as IL-1β and IL-6, thus participating in various inflammatory processes. COX-2 is upregulated in inflammatory conditions and facilitates the conversion of arachidonic acid into prostaglandin H2, which is then transformed into PGE2 by microsomal PGE synthase. The inhibition of the NF-κB signaling pathway leads to decreased COX-2 protein expression (Xu et al., 2020; Yu et al., 2018).

Thus, this study hypothesized that the therapeutic effects of the KHJ formula on acute pharyngitis were mediated by regulating the NF-κB/COX-2 signaling pathway. The experimental results indicated that, in the rat model group, ammonia stimulation significantly increased the expression of p-NF-κB p65/NF-κB p65 and COX-2 proteins in pharyngeal tissues. This suggested that ammonia stimulation led to elevated levels of pro-inflammatory mediators and activation of the NF-κB/COX-2 signaling pathway. In contrast, the expression of p-NF-κB p65/NF-κB p65 and COX-2 proteins was significantly reduced in the treated groups. These findings demonstrate that both high-dose and combined formulations of KHJ components effectively alleviate the inflammatory response in acute pharyngitis by regulating the NF-κB/COX-2 signaling pathway. Moreover, the molecular docking of the quantified HPLC components exhibited significant activity of the components in the tested enzyme active sites, further confirming the results of the *in vivo* study.

**FIGURE 9:**
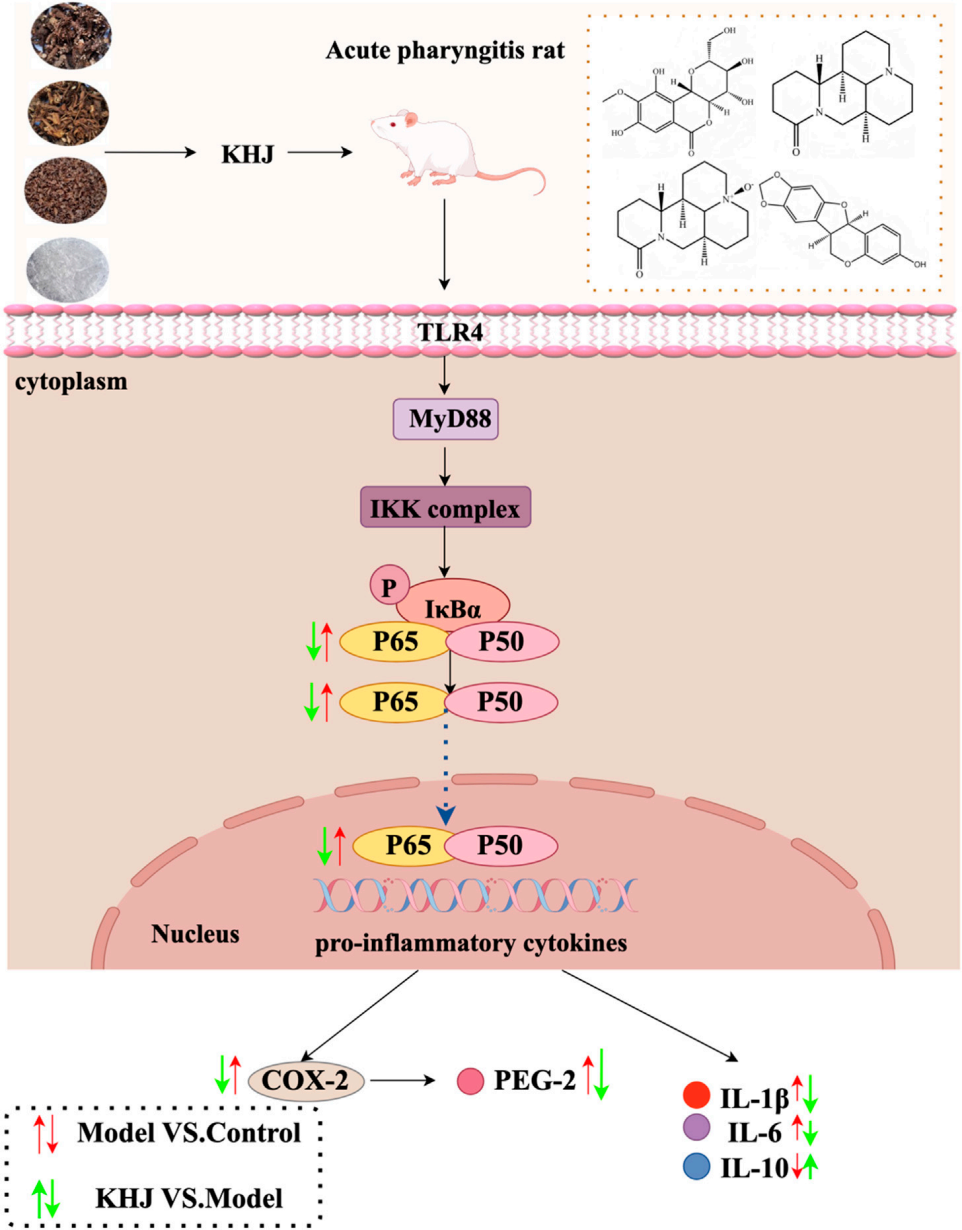
Schematic representation of the underlying mechanisms of KHJ against ammonia-induced acute pharyngitis in rats.

The present study did not directly measure TLR4 or MyD88 levels. However, it is well established that TLR4 recognizes endogenous damage-associated molecular patterns (DAMPs) released from necrotic cells, such as HMGB1 and HSPs (Gong et al., 2020). It is therefore proposed that ammonia-induced mucosal injury releases DAMPs, which subsequently activate the TLR4/MyD88 pathway on immune cells, ultimately triggering NF-κB-driven inflammation (Yan et al., 2024). This inflammatory cascade operates through two principal mechanisms: first, NF-κB activation directly upregulates COX-2 expression, thereby enhancing the synthesis of PGE2, a key mediator of pain, vasodilation, and edema; second, NF-κB activation promotes a cytokine cascade, increasing the production of pro-inflammatory cytokines such as TNF-α, IL-1β, and IL-6, as well as chemokines such as IL-8 (Zhou et al., 2018). These cytokines not only perpetuate local inflammation but also recruit additional immune cells, thereby amplifying and sustaining the inflammatory response. The experimental data demonstrated that KHJ significantly inhibited NF-κB activation, COX-2 expression, and production of these cytokines. This coordinated suppression at mid- and downstream levels supported the conclusion that KHJ exerted its therapeutic effects by targeting the TLR4/MyD88/NF-κB/COX-2 signaling axis. A detailed schematic of this proposed mechanism is provided in Figure 9.

## 5 Conclusion

This study not only confirmed the efficacy of the Miao medicine KHJ in treating acute pharyngitis, but also demonstrated through pharmacological experiments that the complete KHJ formula exhibited significantly superior anti-inflammatory, analgesic, and overall therapeutic effects compared with any of its individual components. This highlighted the synergistic advantage and scientific rationale of Miao medicine compatibility. The mechanistic research revealed that the core action of KHJ was achieved by regulating the NF-κB/COX-2 signaling pathway, thus demonstrating for the first time that the therapeutic effects of KHJ against acute pharyngitis were mediated *via* this specific pathway. These results substantially validated the rationality of Miao medical theory, thereby establishing a robust scientific foundation for the clinical application and further development of KHJ.

However, this study primarily provides preliminary evidence for the association between the efficacy of KHJ and the suppression of the NF-κB/COX-2 pathway. Though supportive, our findings do not establish definitive mechanistic causality nor verify target engagement by specific active ingredients. Therefore, future studies should aim to delineate the complete signaling cascade and validate the direct protein targets of key constituents, such as bergenin and matrine, to establish causal relationships and fully elucidate the multi-target pharmacology of KHJ.

## Data Availability

The original contributions presented in the study are included in the article/supplementary material, further inquiries can be directed to the corresponding author/s.
