# Supplementary figures and images for: Anti-inflammatory and analgesic effects of Kaihoujian throat spray, and therapeutic mechanism in acute pharyngitis: involvement of the NF-κB/COX-2 pathway and formula deconstruction strategy
Source: Front Pharmacol. 2025 Nov 21;16:1687046. doi: 10.3389/fphar.2025.1687046 (PMC12678347; doi:10.3389/fphar.2025.1687046)

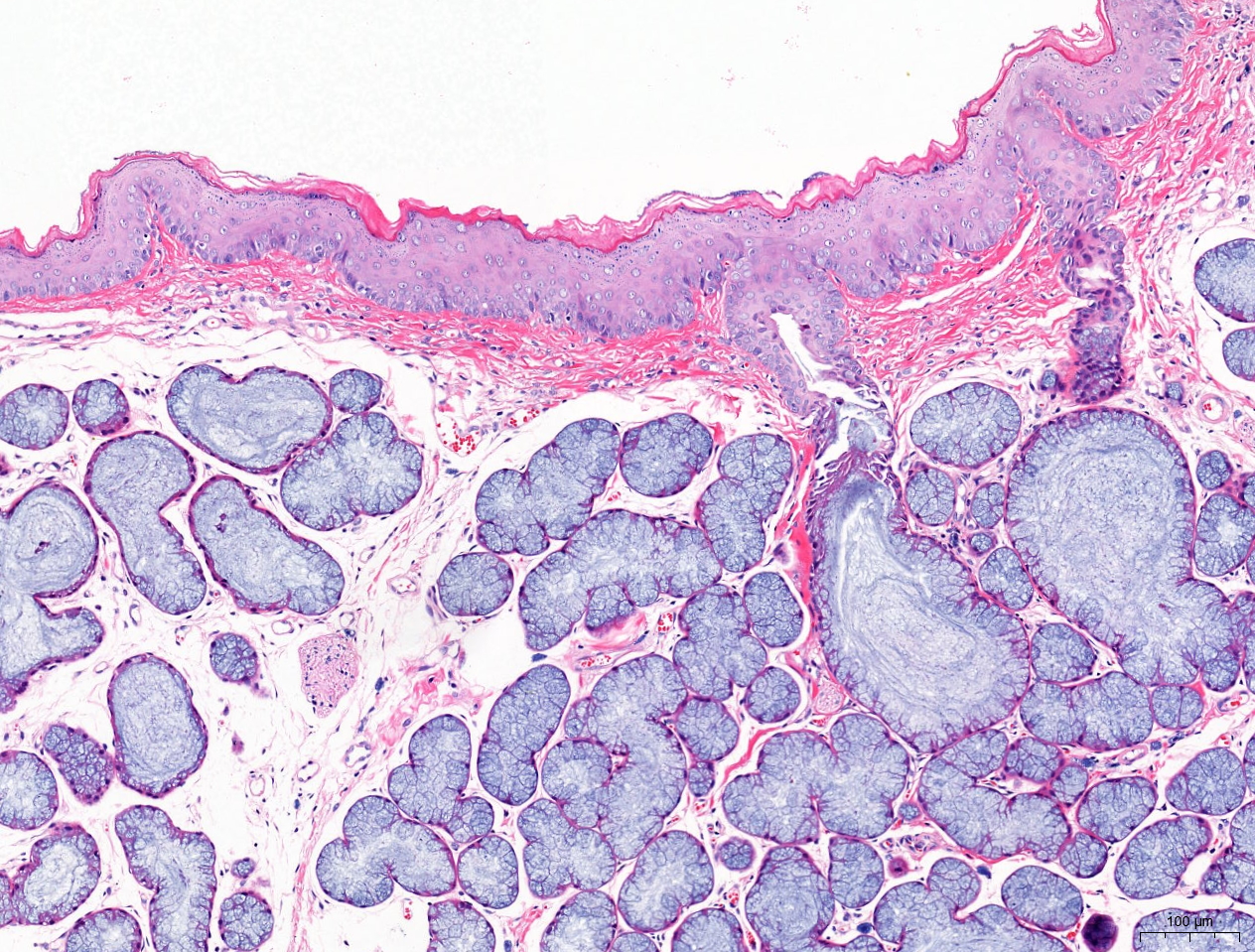

Supplement: Supplementary file 1 [file DataSheet1.zip › Original Images for Blots or Gels or Microscopy/HE staining of rat pharyngeal tissue/A100.jpg]

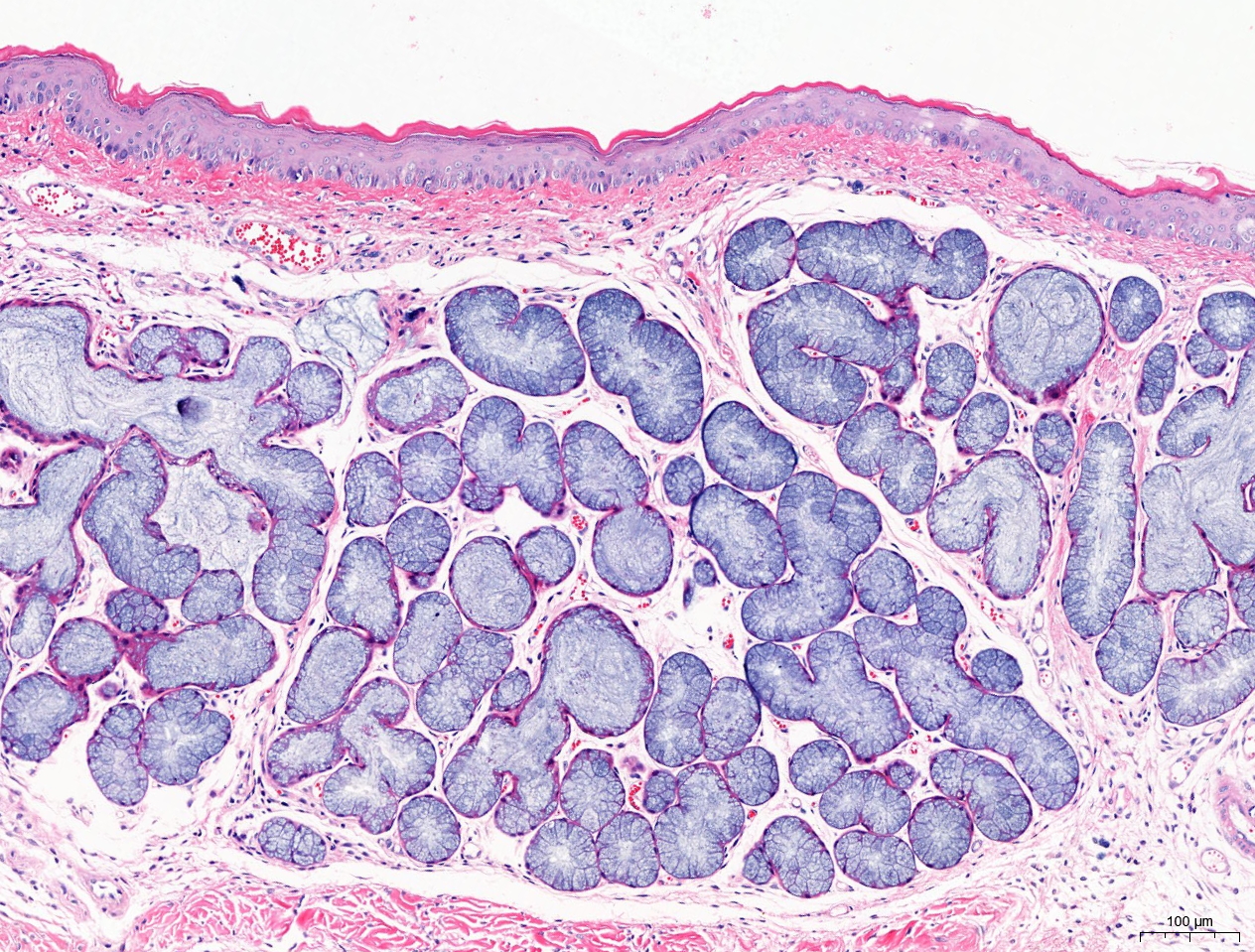

Supplement: Supplementary file 1 [file DataSheet1.zip › Original Images for Blots or Gels or Microscopy/HE staining of rat pharyngeal tissue/AC 100.jpg]

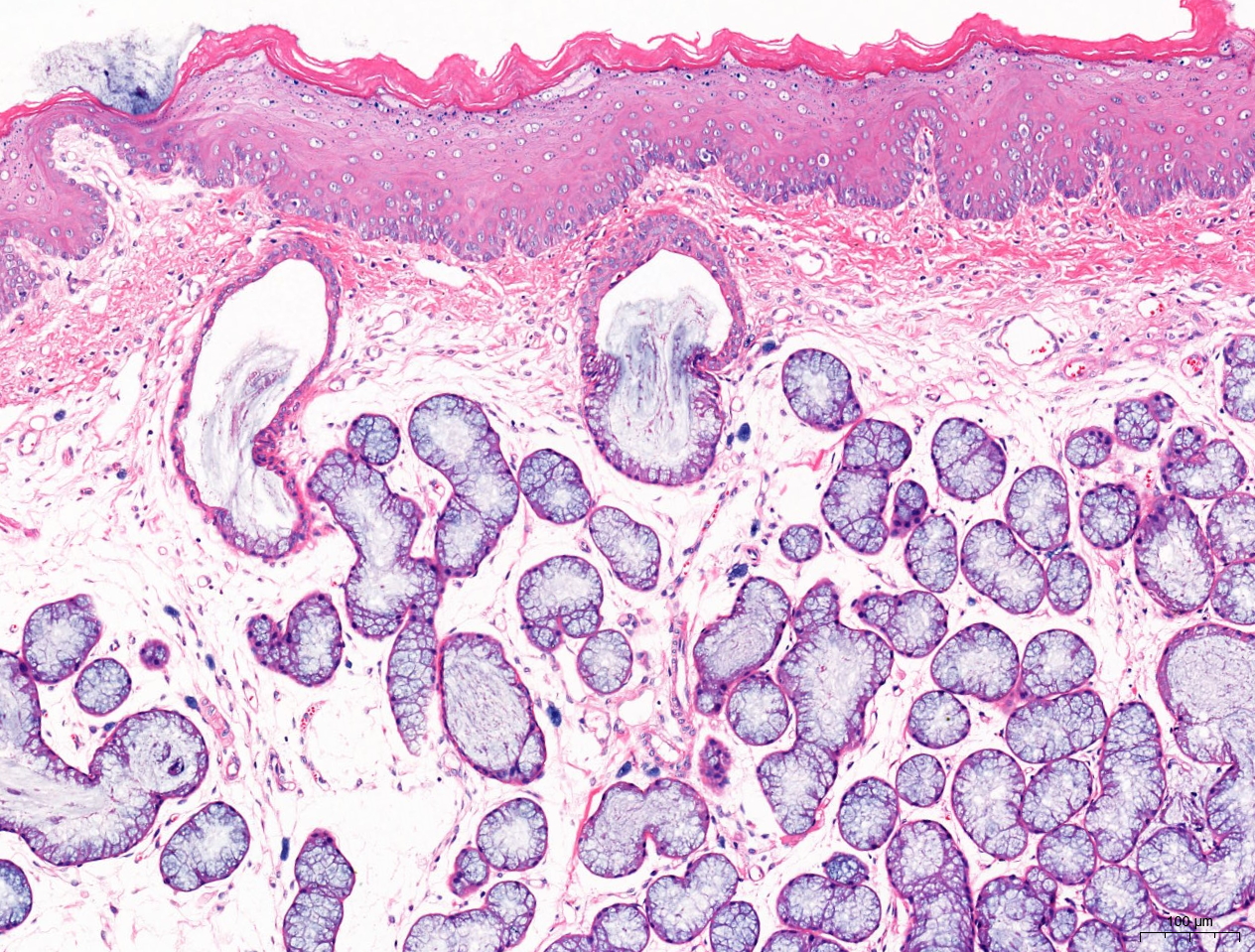

Supplement: Supplementary file 1 [file DataSheet1.zip › Original Images for Blots or Gels or Microscopy/HE staining of rat pharyngeal tissue/AS 100.jpg]

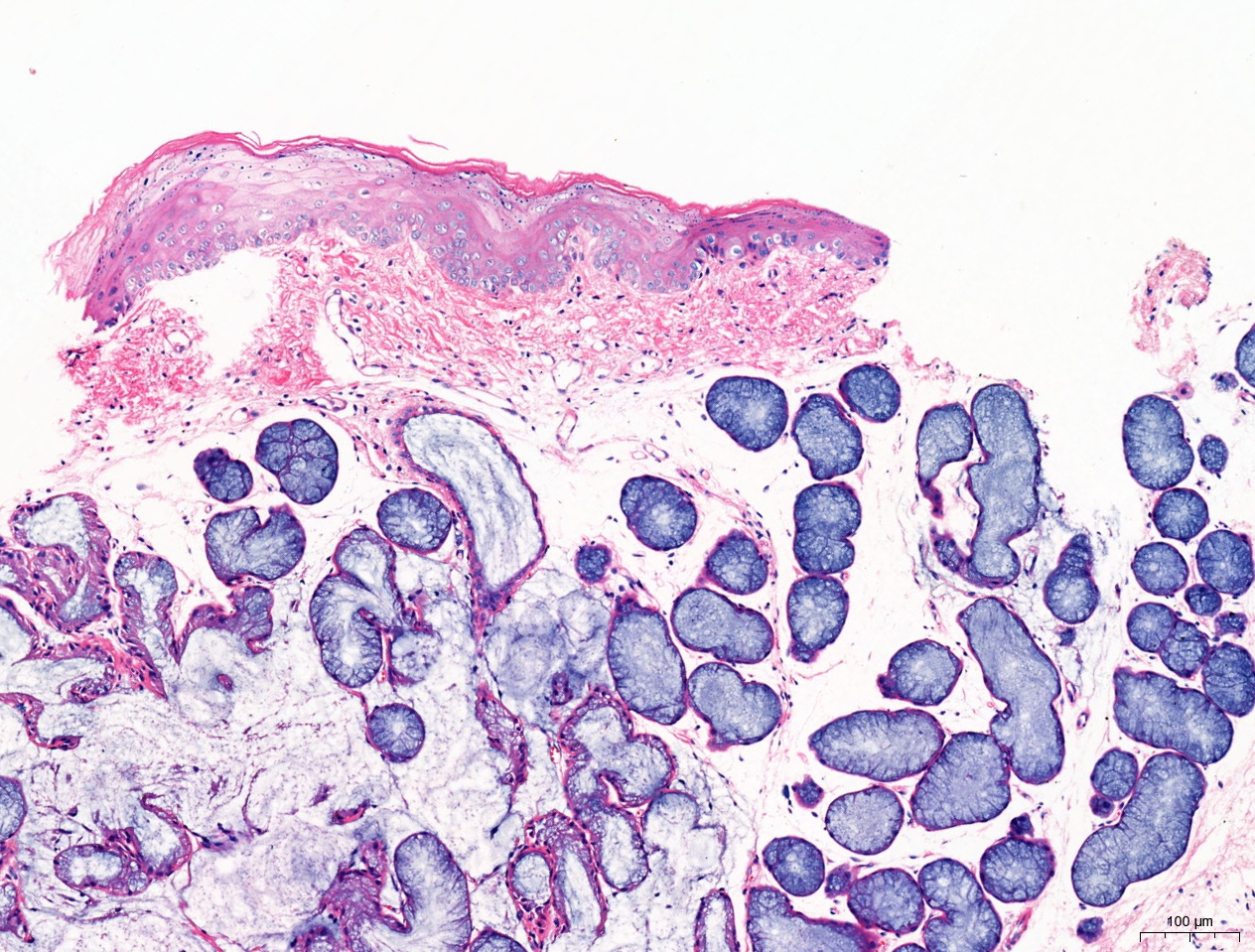

Supplement: Supplementary file 1 [file DataSheet1.zip › Original Images for Blots or Gels or Microscopy/HE staining of rat pharyngeal tissue/Con100.jpg]

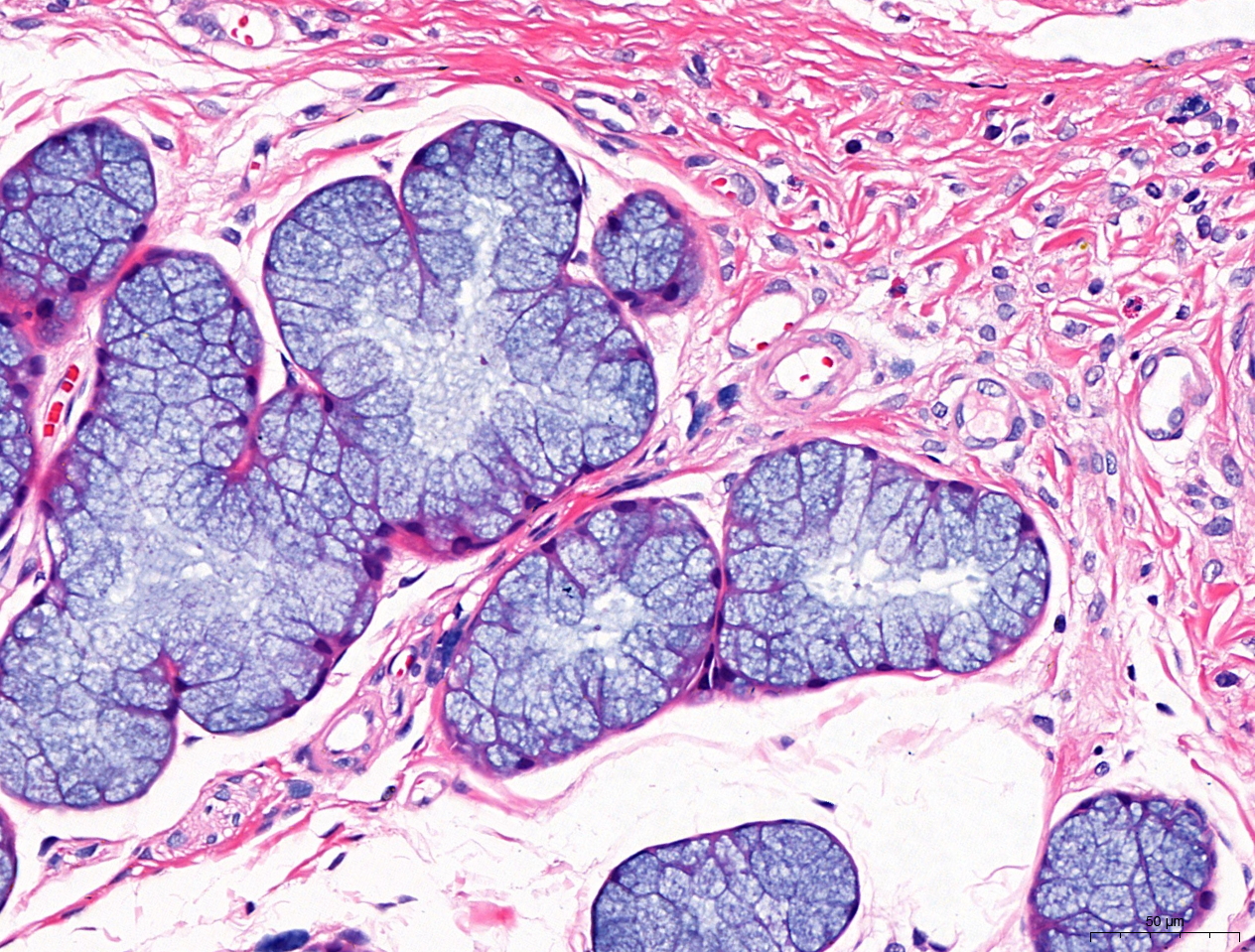

Supplement: Supplementary file 1 [file DataSheet1.zip › Original Images for Blots or Gels or Microscopy/HE staining of rat pharyngeal tissue/KHJ-H.jpg]

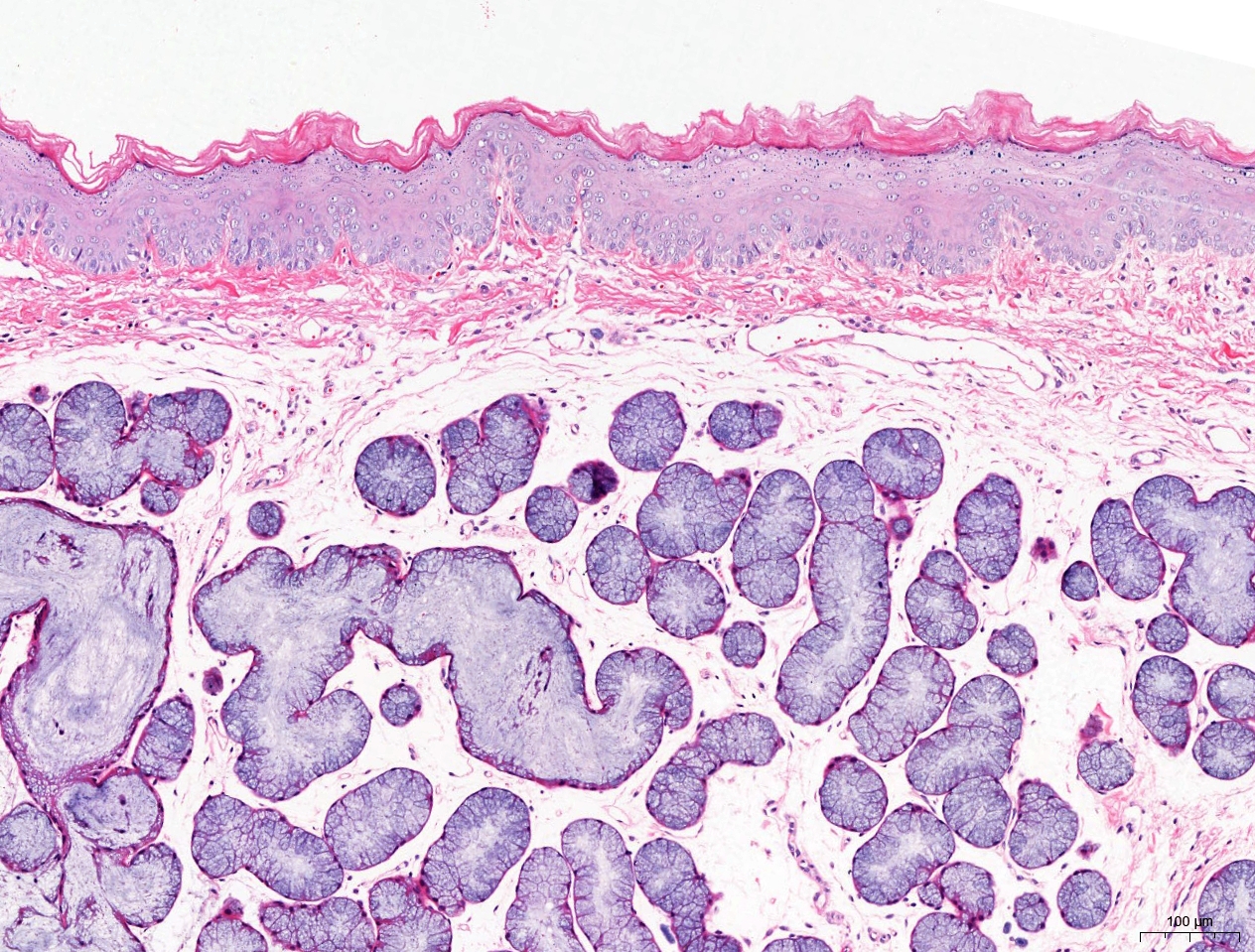

Supplement: Supplementary file 1 [file DataSheet1.zip › Original Images for Blots or Gels or Microscopy/HE staining of rat pharyngeal tissue/KHJ-L100.jpg]

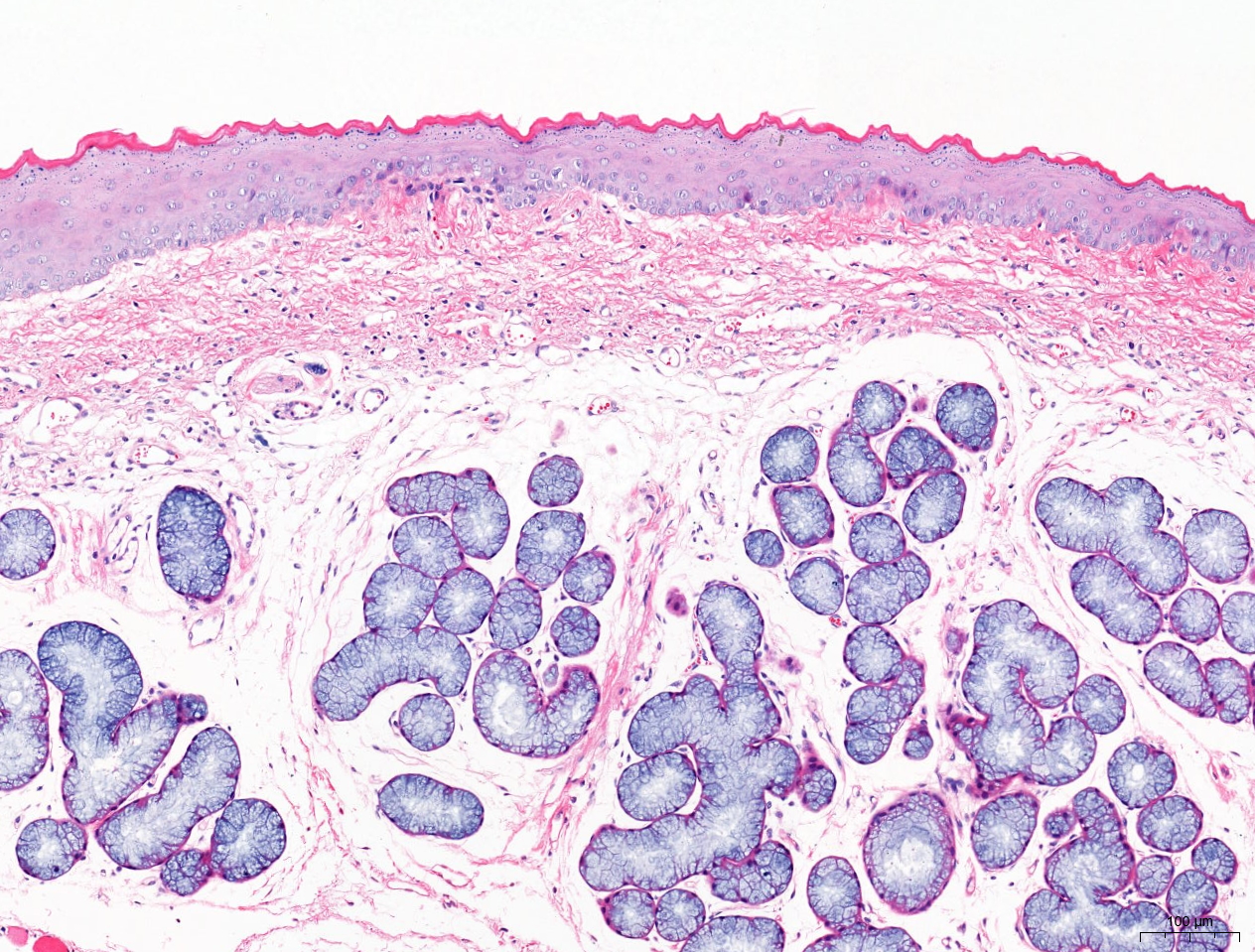

Supplement: Supplementary file 1 [file DataSheet1.zip › Original Images for Blots or Gels or Microscopy/HE staining of rat pharyngeal tissue/KHJ-M100.jpg]

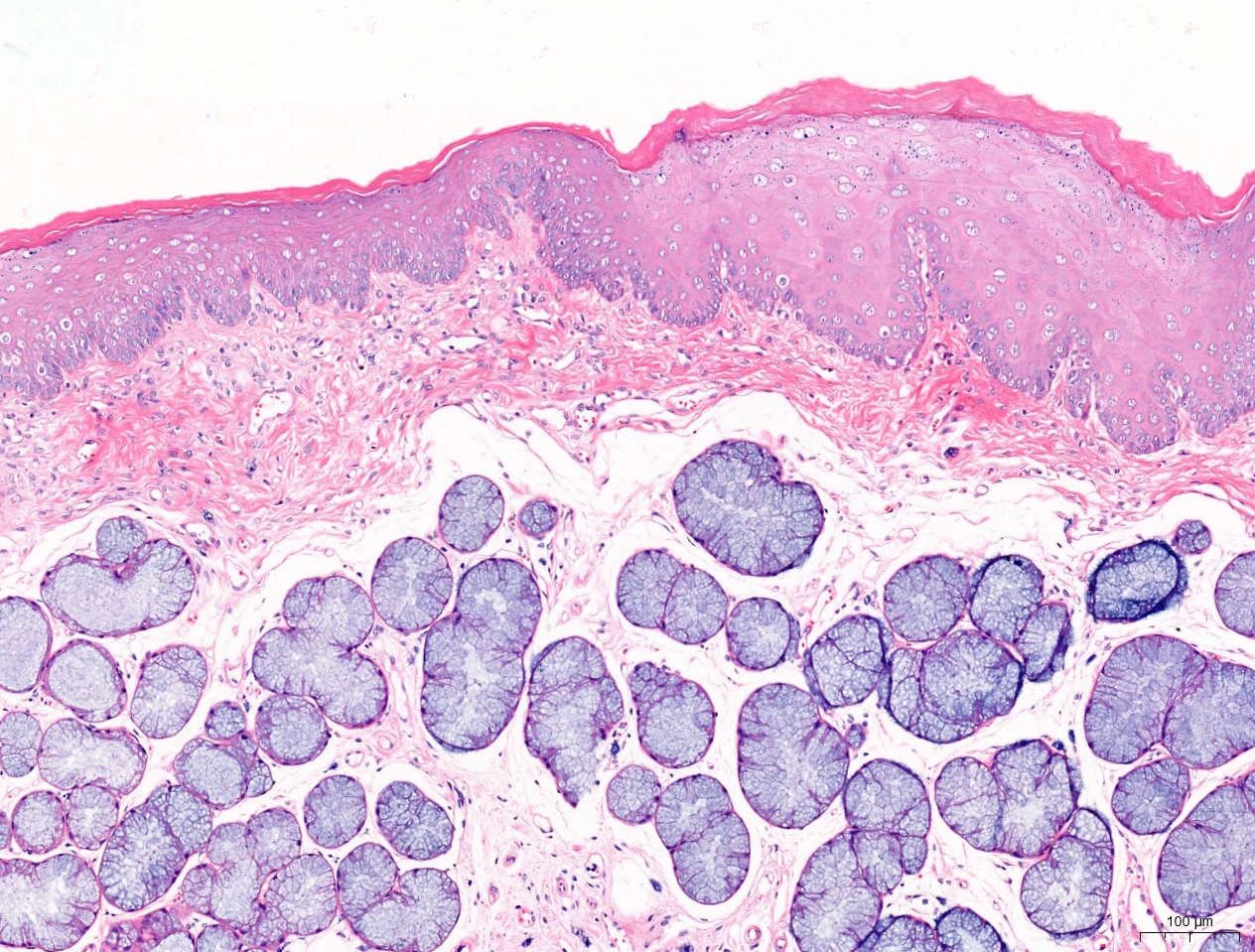

Supplement: Supplementary file 1 [file DataSheet1.zip › Original Images for Blots or Gels or Microscopy/HE staining of rat pharyngeal tissue/MC100.jpg]

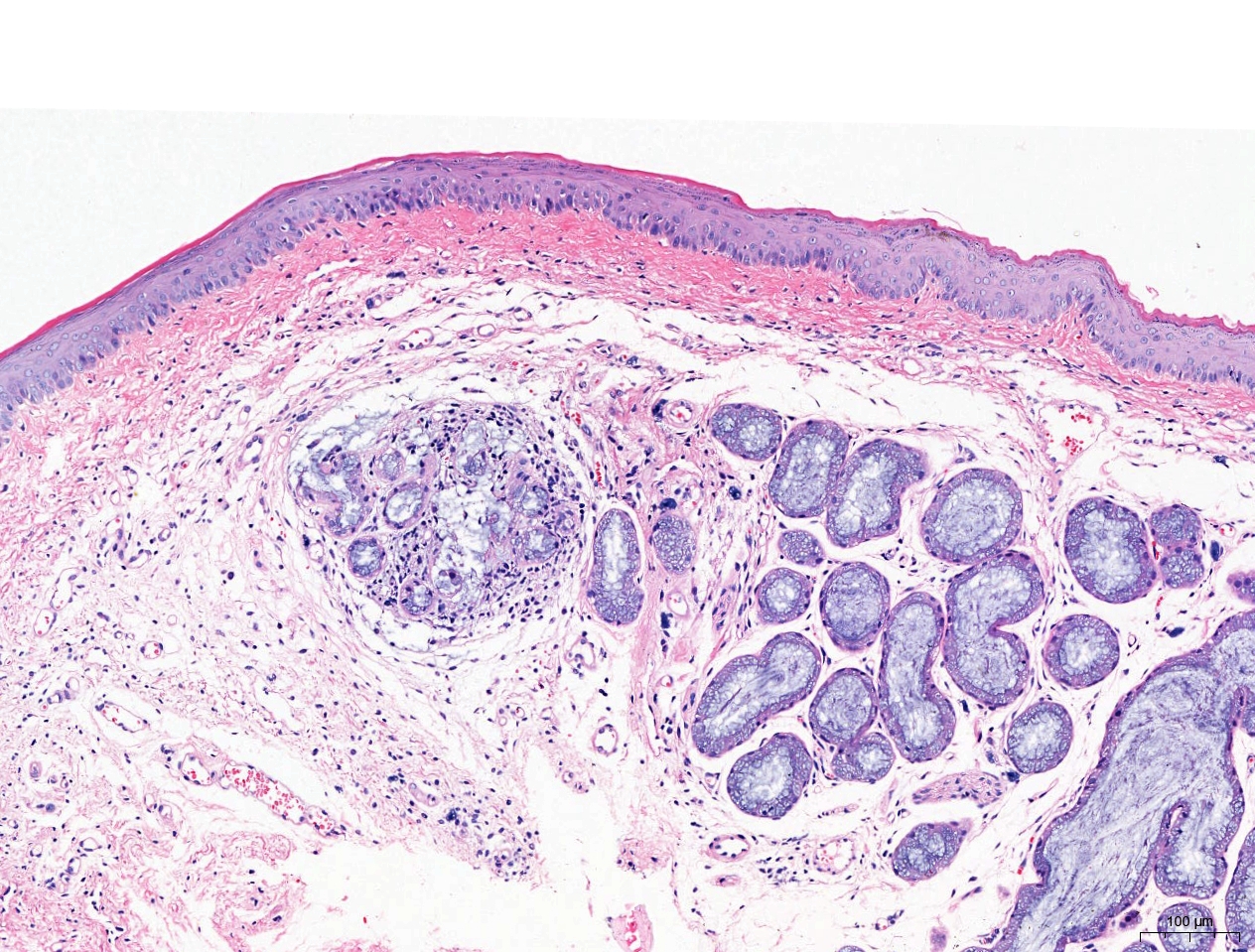

Supplement: Supplementary file 1 [file DataSheet1.zip › Original Images for Blots or Gels or Microscopy/HE staining of rat pharyngeal tissue/Mod100.jpg]

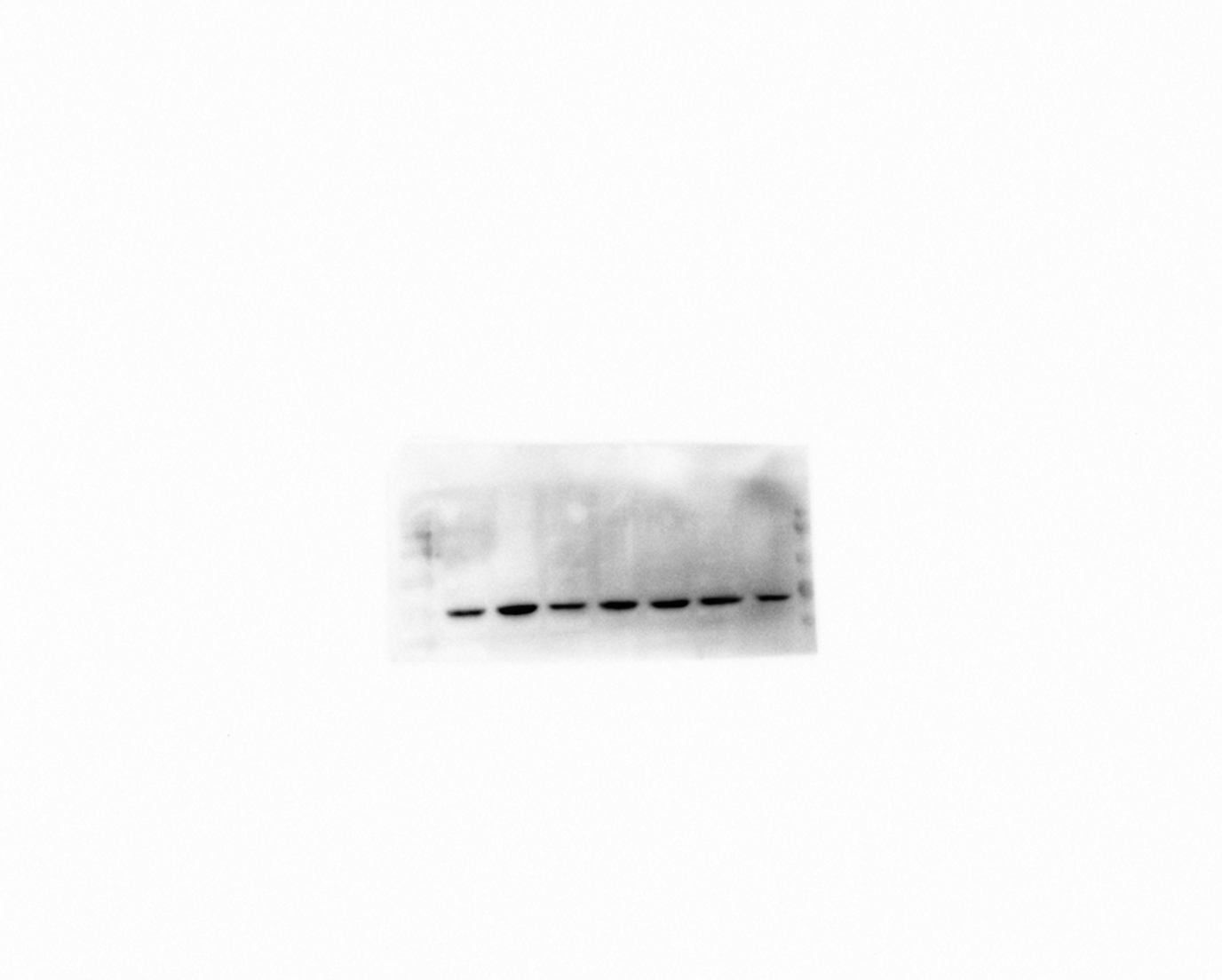

Supplement: Supplementary file 1 [file DataSheet1.zip › Original Images for Blots or Gels or Microscopy/Original Images for Blots/COX-2/COX-2 (1).Tif]

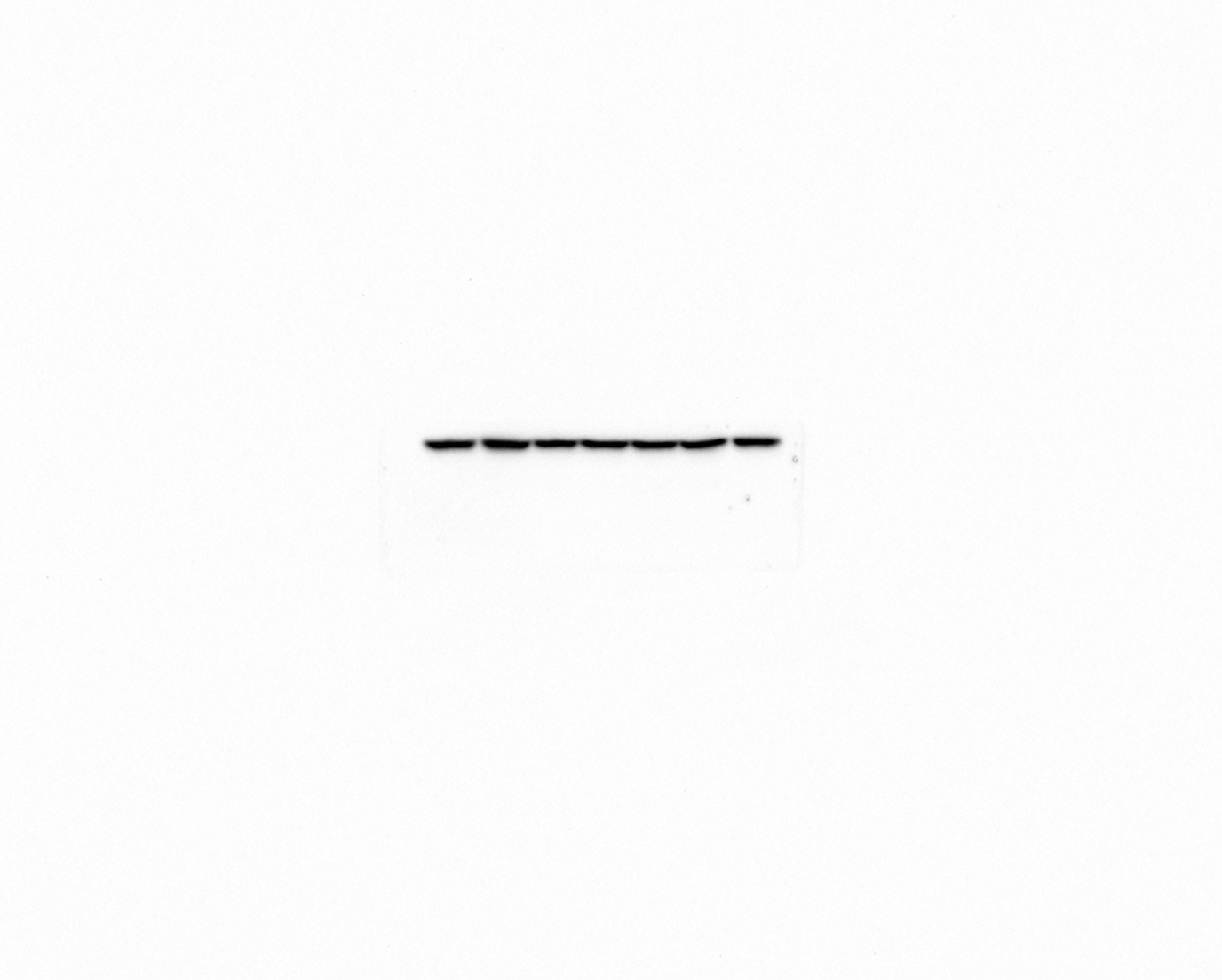

Supplement: Supplementary file 1 [file DataSheet1.zip › Original Images for Blots or Gels or Microscopy/Original Images for Blots/COX-2/COX-2 (2).Tif]

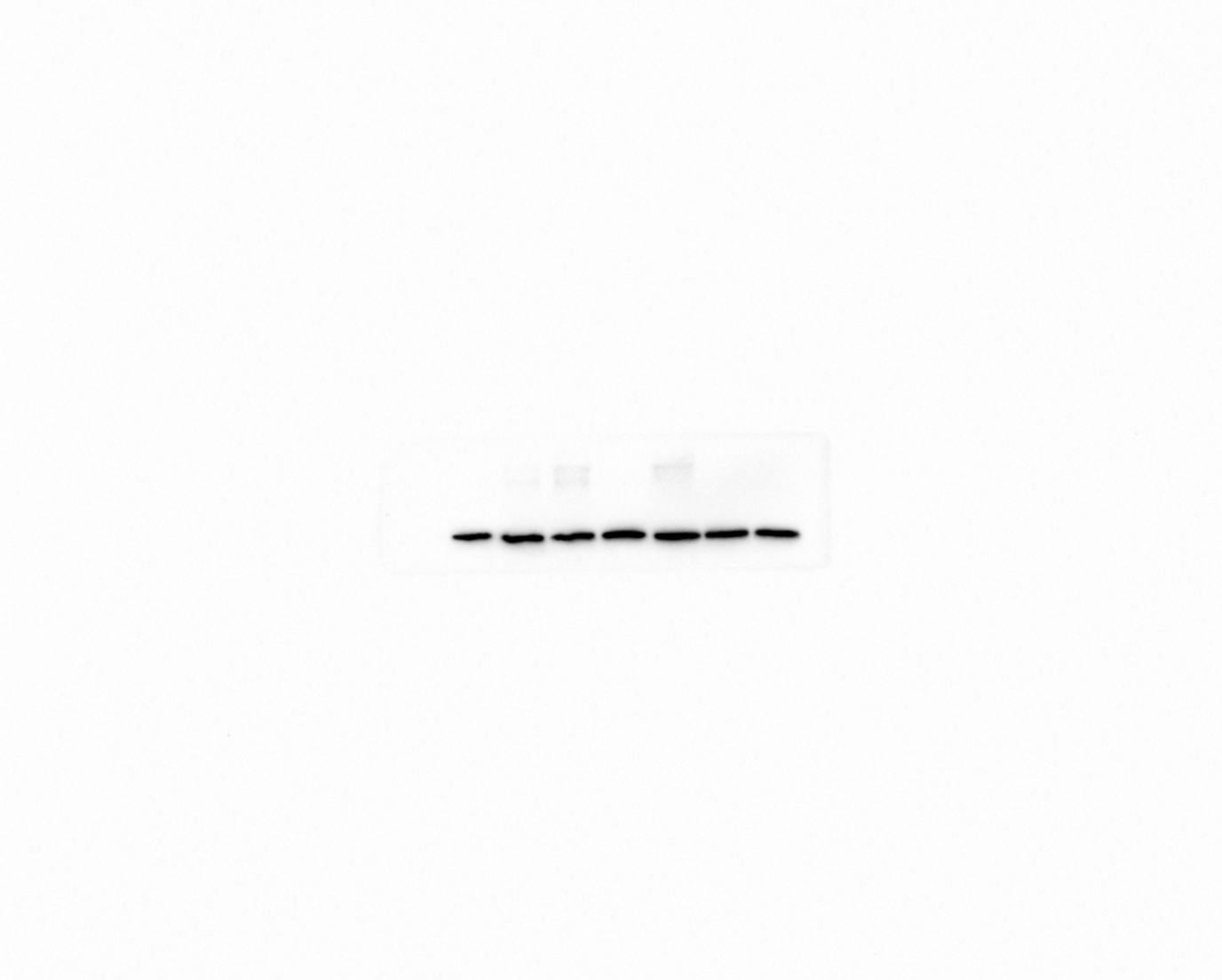

Supplement: Supplementary file 1 [file DataSheet1.zip › Original Images for Blots or Gels or Microscopy/Original Images for Blots/p-p65-p65/NF-kB p65.Tif]

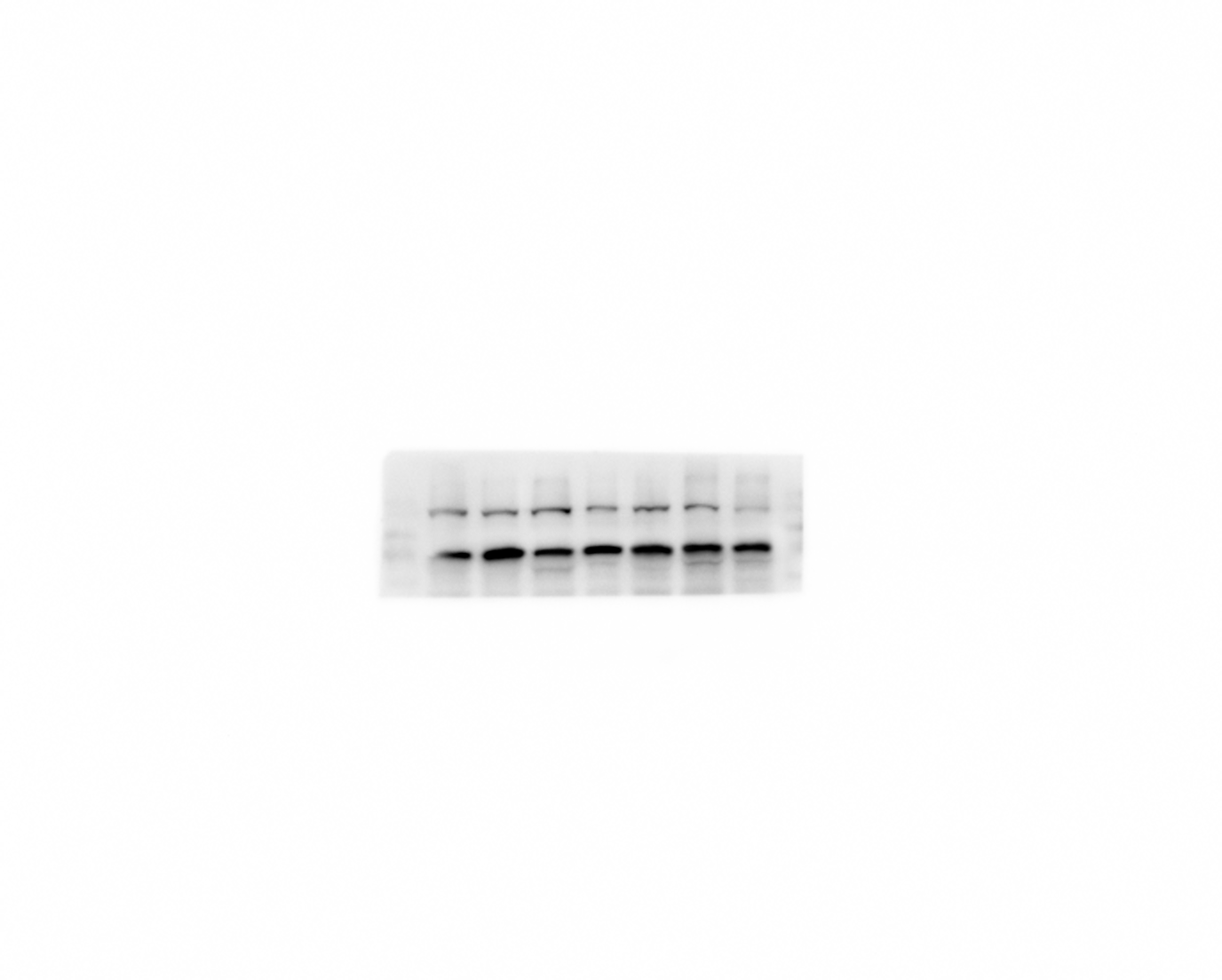

Supplement: Supplementary file 1 [file DataSheet1.zip › Original Images for Blots or Gels or Microscopy/Original Images for Blots/p-p65-p65/p-NF-kB p65.Tif]

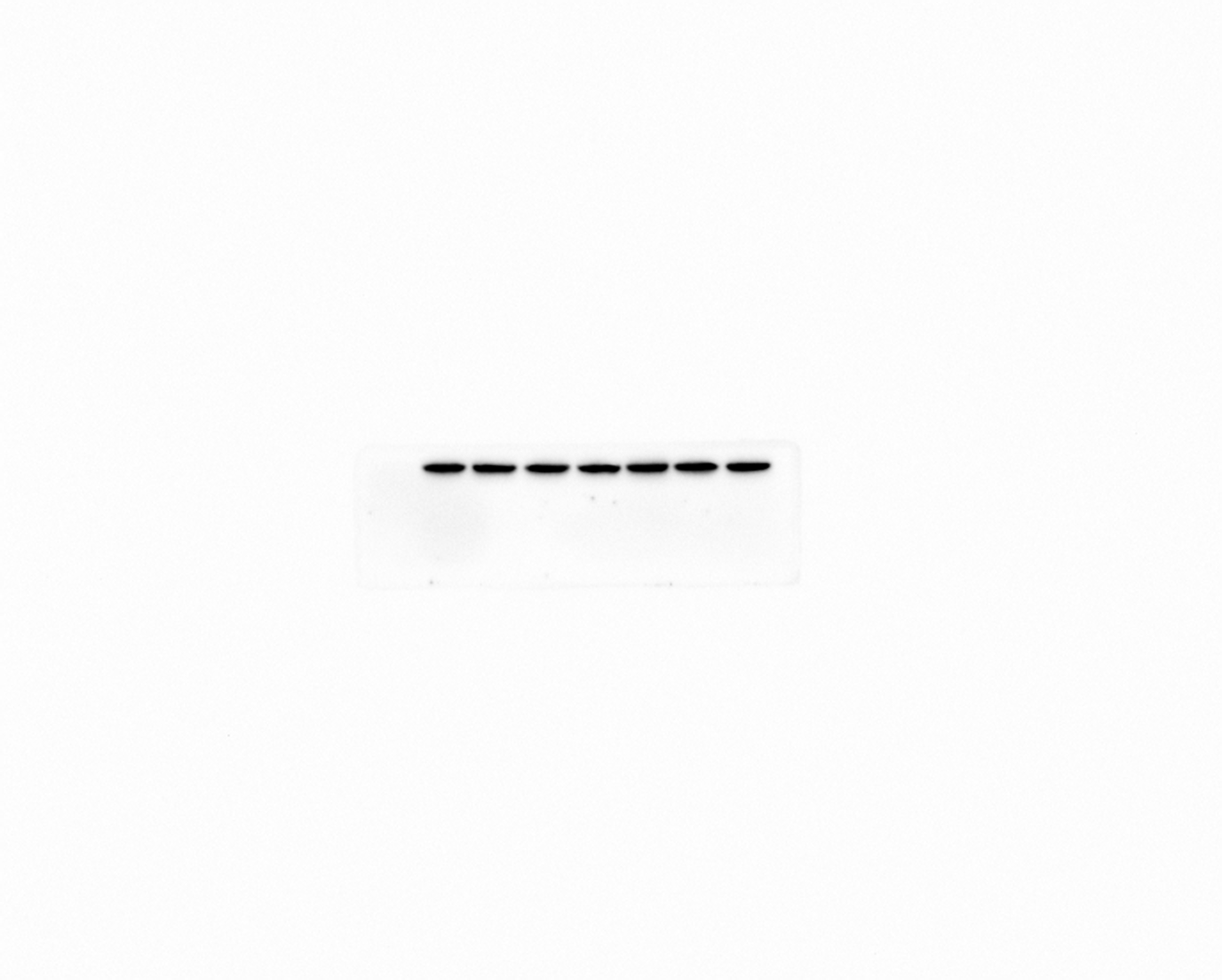

Supplement: Supplementary file 1 [file DataSheet1.zip › Original Images for Blots or Gels or Microscopy/Original Images for Blots/p-p65-p65/β-actin.Tif]
