# Supplementary material for: Anti-inflammatory and analgesic effects of Kaihoujian throat spray, and therapeutic mechanism in acute pharyngitis: involvement of the NF-κB/COX-2 pathway and formula deconstruction strategy
Source: Front Pharmacol. 2025 Nov 21;16:1687046. doi: 10.3389/fphar.2025.1687046 (PMC12678347; doi:10.3389/fphar.2025.1687046)
Supplement: Supplementary file 2 [file DataSheet2.docx]

***Supplementary materials for*：**

**Anti-inflammatory and Analgesic Effects of Kaihoujian Throat Spray, and Therapeutic Mechanism in Acute Pharyngitis: Involvement of the NF-κB/COX-2 Pathway and Formula Deconstruction Strategy**

Hui Shi^a,1^, Jinhe Zhang^a,1^, Liyan Zhang^a^, Xiu Dong^b^, Chang Liu^a^, Xiongwei Liu^a^,Ying Zhou^a*^, Tingting Feng^a*^

^a^ School of Pharmacy, Guizhou University of Traditional Chinese Medicine, Guiyang 550025, China

^b^ Guizhou Sanli Pharmaceutical Co., Ltd., Anshun Guizhou 561100,China

*Corresponding author.

*E-mail addresses:*yingzhou71@126.com (Y. Zhou), fengtingting040@gzy.edu.cn (Tt. Feng).

^1^These authors contributed equally to this work and shared first authorship.

**1.Plant material**

TABLE S1 Source and batch information of medicinal material sample

| Number | *Ardisia crenata* Sims | *Sophora tonkinensis* Gagnep | *Cryptotympana pustulata* Fabricius |
| --- | --- | --- | --- |
| S1 | Chongqing,China | Chongqing,China | Henan,China |
| S2 | Chongqing,China | Chongqing,China | Henan,China |
| S3 | Chongqing,China | Chongqing,China | Henan,China |
| S4 | Guangxi,China | Guangdong,China | Henan,China |
| S5 | Guizhou,China | Guangxi,China | Henan,China |
| S6 | Chongqing,China | Guangxi,China | Henan,China |
| S7 | Chongqing,China | Guangxi,China | Sichuan,China |
| S8 | Guizhou,China | Guizhou,China | Hebei,China |
| S9 | Guizhou,China | Guangxi,China | Sichuan,China |
| S10 | Guizhou,China | Guangxi,China | Sichuan,China |

1. **Fingerprint spectra**


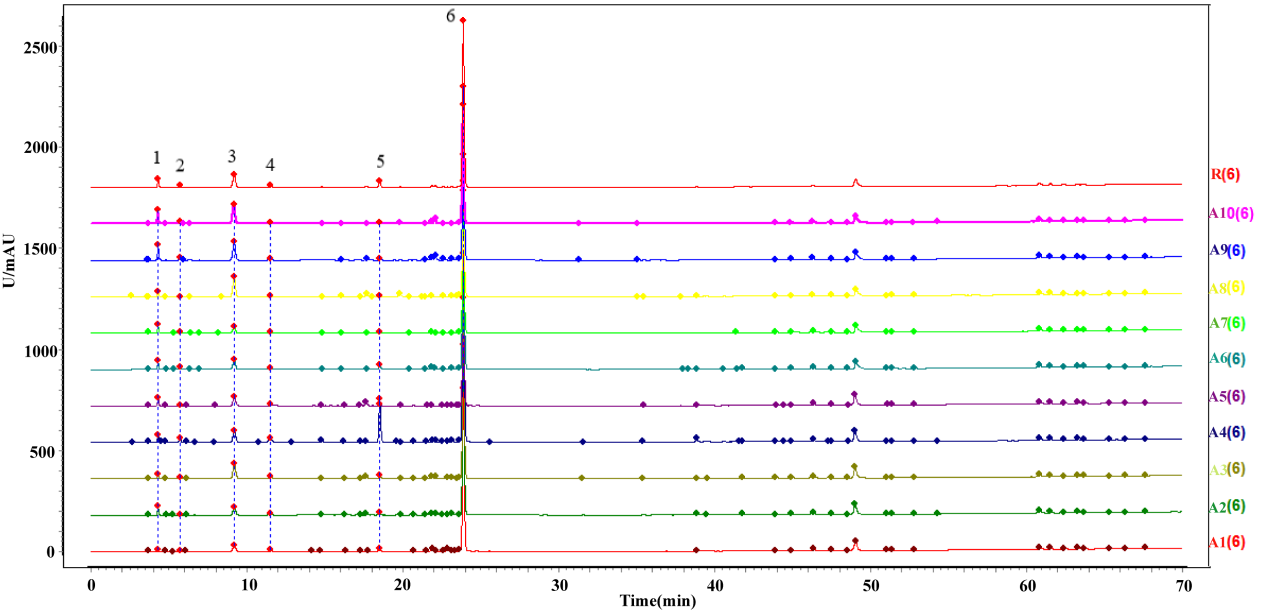


FIGURE 1S 10 batches of fingerprint spectra of A


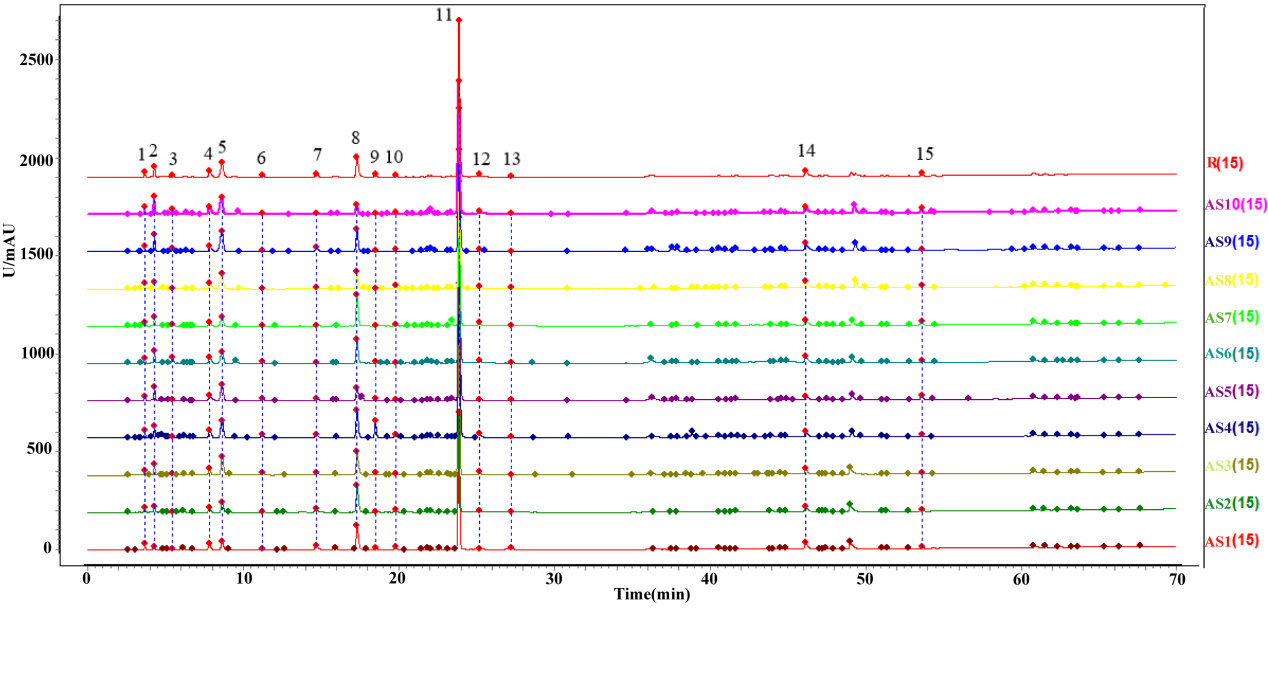


FIGURE 2S 10 batches of fingerprint spectra of AS


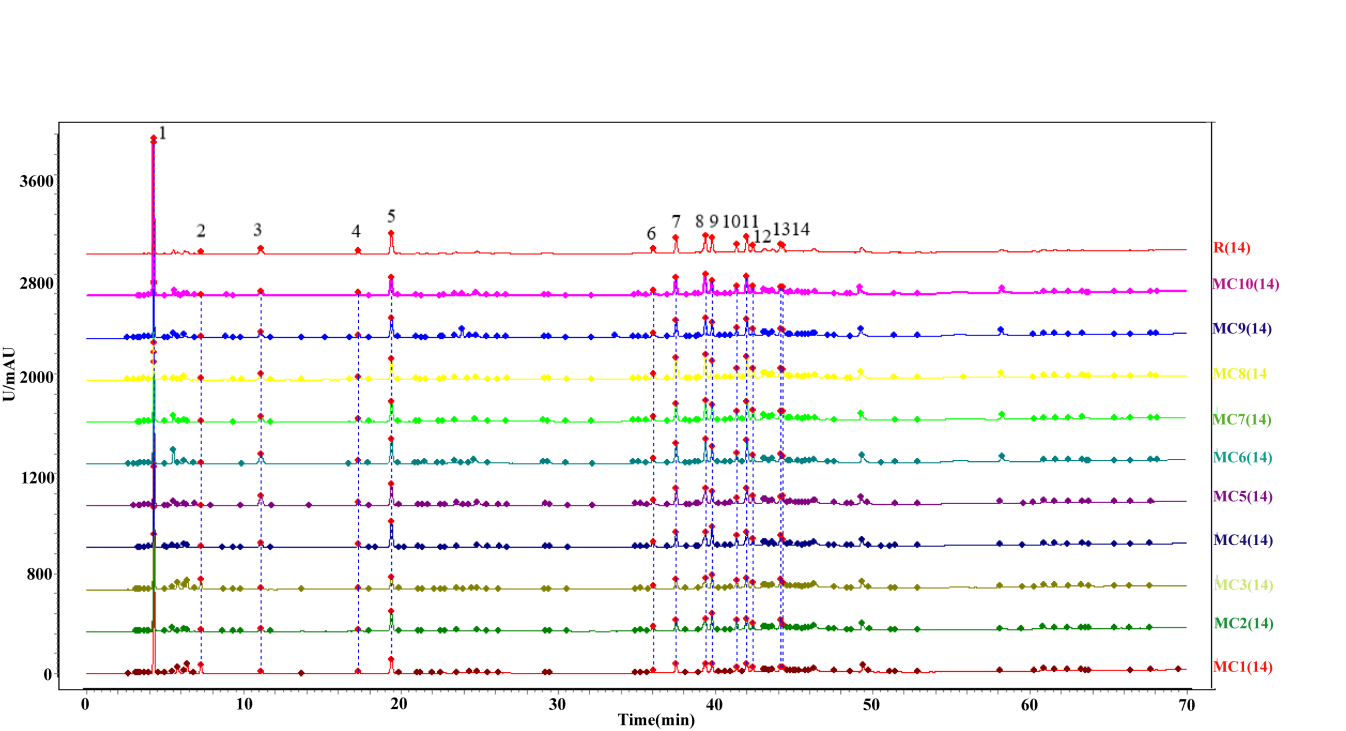


FIGURE3S 10 batches of fingerprint spectra of MC

**3.Similarity Evaluation Results**

TABLE S2-1 Similarity evaluation results for 10 batches of KHJ

| 编号 | S1 | S2 | S3 | S4 | S5 | S6 | S7 | S8 | S9 | S10 | R |
| --- | --- | --- | --- | --- | --- | --- | --- | --- | --- | --- | --- |
| S1 | 1.000 | 0.991 | 0.992 | 0.982 | 0.982 | 0.988 | 0.991 | 0.979 | 0.979 | 0.981 | 0.989 |
| S2 | 0.991 | 1.000 | 0.995 | 0.989 | 0.998 | 0.997 | 0.996 | 0.987 | 0.993 | 0.990 | 0.997 |
| S3 | 0.992 | 0.995 | 1.000 | 0.997 | 0.992 | 0.996 | 0.997 | 0.994 | 0.994 | 0.997 | 0.999 |
| S4 | 0.982 | 0.989 | 0.997 | 1.000 | 0.990 | 0.994 | 0.995 | 0.998 | 0.995 | 0.999 | 0.997 |
| S5 | 0.982 | 0.998 | 0.992 | 0.990 | 1.000 | 0.997 | 0.995 | 0.989 | 0.996 | 0.992 | 0.996 |
| S6 | 0.988 | 0.997 | 0.996 | 0.994 | 0.997 | 1.000 | 0.999 | 0.993 | 0.995 | 0.994 | 0.999 |
| S7 | 0.991 | 0.996 | 0.997 | 0.995 | 0.995 | 0.999 | 1.000 | 0.994 | 0.995 | 0.996 | 0.999 |
| S8 | 0.979 | 0.987 | 0.994 | 0.998 | 0.989 | 0.993 | 0.994 | 1.000 | 0.995 | 0.997 | 0.996 |
| S9 | 0.979 | 0.993 | 0.994 | 0.995 | 0.996 | 0.995 | 0.995 | 0.995 | 1.000 | 0.997 | 0.998 |
| S10 | 0.981 | 0.990 | 0.997 | 0.999 | 0.992 | 0.994 | 0.996 | 0.997 | 0.997 | 1.000 | 0.998 |
| R | 0.989 | 0.997 | 0.999 | 0.997 | 0.996 | 0.999 | 0.999 | 0.996 | 0.998 | 0.998 | 1.000 |

TABLE S2-2 Similarity evaluation results for 10 batches of A

| 编号 | A1 | A2 | A3 | A4 | A5 | A6 | A7 | A8 | A9 | A10 | R |
| --- | --- | --- | --- | --- | --- | --- | --- | --- | --- | --- | --- |
| Z1 | 1.000 | 0.999 | 0.998 | 0.990 | 0.999 | 0.999 | 0.999 | 0.989 | 0.994 | 0.983 | 0.998 |
| Z2 | 0.999 | 1.000 | 0.998 | 0.990 | 1.000 | 1.000 | 1.000 | 0.992 | 0.997 | 0.987 | 0.999 |
| Z3 | 0.998 | 0.998 | 1.000 | 0.988 | 0.999 | 0.999 | 0.998 | 0.997 | 0.999 | 0.993 | 1.000 |
| Z4 | 0.990 | 0.990 | 0.988 | 1.000 | 0.988 | 0.991 | 0.989 | 0.979 | 0.985 | 0.974 | 0.992 |
| Z5 | 0.999 | 1.000 | 0.999 | 0.988 | 1.000 | 1.000 | 1.000 | 0.994 | 0.998 | 0.989 | 0.999 |
| Z6 | 0.999 | 1.000 | 0.999 | 0.991 | 1.000 | 1.000 | 1.000 | 0.992 | 0.997 | 0.988 | 0.999 |
| Z7 | 0.999 | 1.000 | 0.998 | 0.989 | 1.000 | 1.000 | 1.000 | 0.992 | 0.997 | 0.988 | 0.999 |
| Z8 | 0.989 | 0.992 | 0.997 | 0.979 | 0.994 | 0.992 | 0.992 | 1.000 | 0.998 | 0.998 | 0.995 |
| Z9 | 0.994 | 0.997 | 0.999 | 0.985 | 0.998 | 0.997 | 0.997 | 0.998 | 1.000 | 0.997 | 0.999 |
| Z10 | 0.983 | 0.987 | 0.993 | 0.974 | 0.989 | 0.988 | 0.988 | 0.998 | 0.997 | 1.000 | 0.992 |
| R | 0.998 | 0.999 | 1.000 | 0.992 | 0.999 | 0.999 | 0.999 | 0.995 | 0.999 | 0.992 | 1.000 |

TABLE S2-3 Similarity evaluation results for 10 batches of AS

| 编号 | AS1 | AS2 | AS3 | AS4 | AS5 | AS6 | AS7 | AS8 | AS9 | AS10 | R |
| --- | --- | --- | --- | --- | --- | --- | --- | --- | --- | --- | --- |
| ZS1 | 1.000 | 0.996 | 0.993 | 0.991 | 0.984 | 0.994 | 0.994 | 0.993 | 0.991 | 0.982 | 0.996 |
| ZS2 | 0.996 | 1.000 | 0.985 | 0.980 | 0.970 | 0.985 | 0.998 | 0.986 | 0.989 | 0.972 | 0.989 |
| ZS3 | 0.993 | 0.985 | 1.000 | 0.998 | 0.996 | 0.998 | 0.982 | 0.998 | 0.995 | 0.994 | 0.999 |
| ZS4 | 0.991 | 0.980 | 0.998 | 1.000 | 0.996 | 0.997 | 0.978 | 0.995 | 0.989 | 0.989 | 0.997 |
| ZS5 | 0.984 | 0.970 | 0.996 | 0.996 | 1.000 | 0.996 | 0.967 | 0.994 | 0.987 | 0.993 | 0.995 |
| ZS6 | 0.994 | 0.985 | 0.998 | 0.997 | 0.996 | 1.000 | 0.984 | 0.996 | 0.992 | 0.991 | 0.999 |
| ZS7 | 0.994 | 0.998 | 0.982 | 0.978 | 0.967 | 0.984 | 1.000 | 0.983 | 0.985 | 0.969 | 0.987 |
| ZS8 | 0.993 | 0.986 | 0.998 | 0.995 | 0.994 | 0.996 | 0.983 | 1.000 | 0.997 | 0.995 | 0.999 |
| ZS9 | 0.991 | 0.989 | 0.995 | 0.989 | 0.987 | 0.992 | 0.985 | 0.997 | 1.000 | 0.994 | 0.996 |
| ZS10 | 0.982 | 0.972 | 0.994 | 0.989 | 0.993 | 0.991 | 0.969 | 0.995 | 0.994 | 1.000 | 0.994 |
| R | 0.996 | 0.989 | 0.999 | 0.997 | 0.995 | 0.999 | 0.987 | 0.999 | 0.996 | 0.994 | 1.000 |

TABLE S2 -4 Similarity evaluation results for 10 batches of MC

| 编号 | MC1 | MC2 | MC3 | MC4 | MC5 | MC6 | MC7 | MC8 | MC9 | MC10 | R |
| --- | --- | --- | --- | --- | --- | --- | --- | --- | --- | --- | --- |
| C1 | 1.000 | 0.979 | 0.994 | 0.866 | 0.977 | 0.908 | 0.973 | 0.618 | 0.795 | 0.986 | 0.960 |
| C2 | 0.979 | 1.000 | 0.989 | 0.944 | 0.990 | 0.962 | 0.996 | 0.744 | 0.888 | 0.995 | 0.992 |
| C3 | 0.994 | 0.989 | 1.000 | 0.903 | 0.982 | 0.932 | 0.984 | 0.682 | 0.840 | 0.991 | 0.977 |
| C4 | 0.866 | 0.944 | 0.903 | 1.000 | 0.932 | 0.984 | 0.950 | 0.913 | 0.984 | 0.925 | 0.967 |
| C5 | 0.977 | 0.990 | 0.982 | 0.932 | 1.000 | 0.971 | 0.996 | 0.746 | 0.889 | 0.997 | 0.992 |
| C6 | 0.908 | 0.962 | 0.932 | 0.984 | 0.971 | 1.000 | 0.975 | 0.879 | 0.971 | 0.959 | 0.987 |
| C7 | 0.973 | 0.996 | 0.984 | 0.950 | 0.996 | 0.975 | 1.000 | 0.773 | 0.907 | 0.997 | 0.998 |
| C8 | 0.618 | 0.744 | 0.682 | 0.913 | 0.746 | 0.879 | 0.773 | 1.000 | 0.966 | 0.727 | 0.810 |
| C9 | 0.795 | 0.888 | 0.840 | 0.984 | 0.889 | 0.971 | 0.907 | 0.966 | 1.000 | 0.875 | 0.932 |
| C10 | 0.986 | 0.995 | 0.991 | 0.925 | 0.997 | 0.959 | 0.997 | 0.727 | 0.875 | 1.000 | 0.990 |
| R | 0.960 | 0.992 | 0.977 | 0.967 | 0.992 | 0.987 | 0.998 | 0.810 | 0.932 | 0.990 | 1.000 |

**4.Validation of fingerprint evaluation method**

##### Precision

TABLE S3-1 The relative retention time of the precision of KHJ

| Number | 1 | 2 | 3 | 4 | 5 | 6 | RSD/% |
| --- | --- | --- | --- | --- | --- | --- | --- |
| Peak 1 | 0.1559 | 0.1568 | 0.1569 | 0.1569 | 0.1569 | 0.1569 | 0.26 |
| Peak 2 | 0.1785 | 0.1799 | 0.1802 | 0.1802 | 0.1804 | 0.1804 | 0.40 |
| Peak 3 | 0.2327 | 0.2361 | 0.2362 | 0.2363 | 0.2366 | 0.2366 | 0.64 |
| Peak 4 | 0.3261 | 0.3311 | 0.3306 | 0.3301 | 0.3299 | 0.3295 | 0.54 |
| Peak 5 | 0.3668 | 0.3714 | 0.3714 | 0.3715 | 0.3721 | 0.3720 | 0.54 |
| Peak 6 | 0.6097 | 0.6183 | 0.6170 | 0.6165 | 0.6162 | 0.6157 | 0.49 |
| Peak 7 | 0.7117 | 0.7219 | 0.7221 | 0.7227 | 0.7232 | 0.7235 | 0.63 |
| Peak 8 | 0.7597 | 0.7649 | 0.7646 | 0.7648 | 0.7649 | 0.7651 | 0.28 |
| Peak 9 | 0.7917 | 0.8014 | 0.8010 | 0.8012 | 0.8015 | 0.8018 | 0.50 |
| Peak 10 | 0.8139 | 0.8219 | 0.8216 | 0.8216 | 0.8217 | 0.8218 | 0.39 |
| Peak 11 | 1.0000 | 1.0000 | 1.0000 | 1.0000 | 1.0000 | 1.0000 | 0.00 |
| Peak 12 | 1.0620 | 1.0650 | 1.0655 | 1.0659 | 1.0662 | 1.0667 | 0.16 |
| Peak 13 | 1.1472 | 1.1473 | 1.1477 | 1.1480 | 1.1479 | 1.1481 | 0.03 |
| Peak 14 | 1.5851 | 1.5808 | 1.5813 | 1.5817 | 1.5813 | 1.5813 | 0.10 |
| Peak 15 | 1.6669 | 1.6622 | 1.6626 | 1.6629 | 1.6625 | 1.6625 | 0.11 |
| Peak 16 | 1.6849 | 1.6797 | 1.6801 | 1.6805 | 1.6801 | 1.6801 | 0.12 |
| Peak 17 | 1.7822 | 1.7765 | 1.7771 | 1.7772 | 1.7768 | 1.7767 | 0.12 |
| Peak 18 | 1.8029 | 1.7967 | 1.7974 | 1.7973 | 1.7970 | 1.7968 | 0.13 |
| Peak 19 | 1.8731 | 1.8667 | 1.8674 | 1.8684 | 1.8680 | 1.8669 | 0.13 |
| Peak 20 | 1.8851 | 1.8782 | 1.8790 | 1.8789 | 1.8786 | 1.8784 | 0.14 |
| Peak 21 | 1.9627 | 1.9558 | 1.9566 | 1.9566 | 1.9563 | 1.9560 | 0.14 |
| Peak 22 | 2.2718 | 2.2644 | 2.2656 | 2.2656 | 2.2652 | 2.2649 | 0.12 |

TABLE S3-2 The relative peak area of the precision of KHJ

| Number | 1 | 2 | 3 | 4 | 5 | 6 | RSD/% |
| --- | --- | --- | --- | --- | --- | --- | --- |
| Peak 1 | 0.0221 | 0.0215 | 0.0215 | 0.0217 | 0.0217 | 0.0217 | 1.01 |
| Peak 2 | 0.1657 | 0.1640 | 0.1640 | 0.1653 | 0.1661 | 0.1634 | 0.66 |
| Peak 3 | 0.0172 | 0.0172 | 0.0174 | 0.0174 | 0.0171 | 0.0175 | 0.90 |
| Peak 4 | 0.0834 | 0.0840 | 0.0837 | 0.0841 | 0.0838 | 0.0842 | 0.35 |
| Peak 5 | 0.3119 | 0.3102 | 0.3100 | 0.3061 | 0.3069 | 0.3099 | 0.71 |
| Peak 6 | 0.0426 | 0.0414 | 0.0416 | 0.0416 | 0.0421 | 0.0420 | 1.05 |
| Peak 7 | 0.2300 | 0.2236 | 0.2231 | 0.2232 | 0.2229 | 0.2227 | 1.26 |
| Peak 8 | 0.0140 | 0.0137 | 0.0135 | 0.0135 | 0.0137 | 0.0135 | 1.45 |
| Peak 9 | 0.0258 | 0.0272 | 0.0272 | 0.0272 | 0.0273 | 0.0272 | 2.15 |
| Peak 10 | 0.0358 | 0.0351 | 0.0352 | 0.0352 | 0.0353 | 0.0352 | 0.72 |
| Peak 11 | 1.0000 | 1.0000 | 1.0000 | 1.0000 | 1.0000 | 1.0000 | 0.00 |
| Peak 12 | 0.0223 | 0.0216 | 0.0219 | 0.0217 | 0.0218 | 0.0217 | 1.15 |
| Peak 13 | 0.0223 | 0.0213 | 0.0211 | 0.0210 | 0.0211 | 0.0211 | 2.31 |
| Peak 14 | 0.0295 | 0.0288 | 0.0286 | 0.0285 | 0.0286 | 0.0285 | 1.33 |
| Peak 15 | 0.0256 | 0.0259 | 0.0262 | 0.0265 | 0.0265 | 0.0267 | 1.59 |
| Peak 16 | 0.0119 | 0.0124 | 0.0125 | 0.0124 | 0.0125 | 0.0125 | 1.89 |
| Peak 17 | 0.0207 | 0.0199 | 0.0201 | 0.0200 | 0.0212 | 0.0211 | 2.81 |
| Peak 18 | 0.0132 | 0.0131 | 0.0131 | 0.0131 | 0.0129 | 0.0130 | 0.79 |
| Peak 19 | 0.0074 | 0.0076 | 0.0075 | 0.0076 | 0.0076 | 0.0076 | 1.11 |
| Peak 20 | 0.0094 | 0.0096 | 0.0096 | 0.0096 | 0.0095 | 0.0096 | 0.88 |
| Peak 21 | 0.0491 | 0.0502 | 0.0501 | 0.0500 | 0.0500 | 0.0492 | 0.97 |
| Peak 22 | 0.0308 | 0.0302 | 0.0302 | 0.0301 | 0.0301 | 0.0299 | 1.01 |

##### Repeatability

TABLE S3-3 The relative retention time of the repeatability of KHJ

| Number | 1 | 2 | 3 | 4 | 5 | 6 | RSD/% |
| --- | --- | --- | --- | --- | --- | --- | --- |
| Peak 1 | 0.1561 | 0.1565 | 0.1566 | 0.1567 | 0.1565 | 0.1565 | 0.13 |
| Peak 2 | 0.1805 | 0.1811 | 0.1812 | 0.1813 | 0.1812 | 0.1812 | 0.16 |
| Peak 3 | 0.2367 | 0.2370 | 0.2368 | 0.2372 | 0.2369 | 0.2368 | 0.08 |
| Peak 4 | 0.3292 | 0.3292 | 0.3288 | 0.3289 | 0.3280 | 0.3283 | 0.15 |
| Peak 5 | 0.3722 | 0.3715 | 0.3706 | 0.3717 | 0.3707 | 0.3706 | 0.18 |
| Peak 6 | 0.6154 | 0.6144 | 0.6133 | 0.6141 | 0.6126 | 0.6128 | 0.17 |
| Peak 7 | 0.7238 | 0.7231 | 0.7225 | 0.7233 | 0.7232 | 0.7230 | 0.06 |
| Peak 8 | 0.7652 | 0.7641 | 0.7634 | 0.7637 | 0.7637 | 0.7636 | 0.09 |
| Peak 9 | 0.8019 | 0.8004 | 0.7994 | 0.8000 | 0.7999 | 0.7998 | 0.11 |
| Peak 10 | 0.8218 | 0.8210 | 0.8203 | 0.8207 | 0.8206 | 0.8205 | 0.07 |
| Peak 11 | 1.0000 | 1.0000 | 1.0000 | 1.0000 | 1.0000 | 1.0000 | 0.00 |
| Peak 12 | 1.0670 | 1.0651 | 1.0652 | 1.0649 | 1.0655 | 1.0654 | 0.07 |
| Peak 13 | 1.1481 | 1.1475 | 1.1479 | 1.1476 | 1.1477 | 1.1477 | 0.02 |
| Peak 14 | 1.5810 | 1.5818 | 1.5823 | 1.5823 | 1.5827 | 1.5822 | 0.04 |
| Peak 15 | 1.6621 | 1.6638 | 1.6643 | 1.6643 | 1.6647 | 1.6641 | 0.06 |
| Peak 16 | 1.6797 | 1.6814 | 1.6819 | 1.6819 | 1.6823 | 1.6817 | 0.05 |
| Peak 17 | 1.7763 | 1.7788 | 1.7794 | 1.7792 | 1.7796 | 1.7790 | 0.07 |
| Peak 18 | 1.7964 | 1.7994 | 1.8000 | 1.7999 | 1.8003 | 1.7996 | 0.08 |
| Peak 19 | 1.8664 | 1.8693 | 1.8698 | 1.8696 | 1.8703 | 1.8695 | 0.07 |
| Peak 20 | 1.8780 | 1.8814 | 1.8819 | 1.8819 | 1.8823 | 1.8816 | 0.08 |
| Peak 21 | 1.9554 | 1.9587 | 1.9593 | 1.9591 | 1.9596 | 1.9590 | 0.08 |
| Peak 22 | 2.2642 | 2.2677 | 2.2683 | 2.2673 | 2.2689 | 2.2680 | 0.07 |

TABLE S3-4 The relative peak area of the repeatability of KHJ

| Number | 1 | 2 | 3 | 4 | 5 | 6 | RSD/% |
| --- | --- | --- | --- | --- | --- | --- | --- |
| Peak 1 | 0.0216 | 0.0219 | 0.0221 | 0.0224 | 0.0227 | 0.0226 | 1.92 |
| Peak 2 | 0.1639 | 0.1619 | 0.1619 | 0.1619 | 0.1627 | 0.1627 | 0.49 |
| Peak 3 | 0.0171 | 0.0175 | 0.0173 | 0.0176 | 0.0178 | 0.0175 | 1.39 |
| Peak 4 | 0.0837 | 0.0844 | 0.0846 | 0.0845 | 0.0850 | 0.0851 | 0.59 |
| Peak 5 | 0.3099 | 0.3102 | 0.3102 | 0.3130 | 0.3131 | 0.3136 | 0.56 |
| Peak 6 | 0.0424 | 0.0427 | 0.0429 | 0.0432 | 0.0434 | 0.0432 | 0.87 |
| Peak 7 | 0.2224 | 0.2256 | 0.2265 | 0.2271 | 0.2270 | 0.2265 | 0.78 |
| Peak 8 | 0.0135 | 0.0135 | 0.0135 | 0.0135 | 0.0135 | 0.0136 | 0.30 |
| Peak 9 | 0.0273 | 0.0274 | 0.0274 | 0.0276 | 0.0274 | 0.0275 | 0.38 |
| Peak 10 | 0.0352 | 0.0355 | 0.0355 | 0.0358 | 0.0357 | 0.0358 | 0.65 |
| Peak 11 | 1.0000 | 1.0000 | 1.0000 | 1.0000 | 1.0000 | 1.0000 | 0.00 |
| Peak 12 | 0.0181 | 0.0183 | 0.0180 | 0.0181 | 0.0182 | 0.0182 | 0.58 |
| Peak 13 | 0.0210 | 0.0208 | 0.0207 | 0.0205 | 0.0198 | 0.0199 | 2.41 |
| Peak 14 | 0.0287 | 0.0293 | 0.0292 | 0.0297 | 0.0294 | 0.0295 | 1.16 |
| Peak 15 | 0.0277 | 0.0293 | 0.0293 | 0.0298 | 0.0298 | 0.0297 | 2.74 |
| Peak 16 | 0.0130 | 0.0138 | 0.0136 | 0.0141 | 0.0138 | 0.0137 | 2.69 |
| Peak 17 | 0.0213 | 0.0204 | 0.0203 | 0.0206 | 0.0203 | 0.0204 | 1.87 |
| Peak 18 | 0.0131 | 0.0131 | 0.0130 | 0.0133 | 0.0131 | 0.0132 | 0.79 |
| Peak 19 | 0.0086 | 0.0085 | 0.0084 | 0.0087 | 0.0086 | 0.0086 | 1.21 |
| Peak 20 | 0.0111 | 0.0112 | 0.0110 | 0.0112 | 0.0113 | 0.0112 | 0.92 |
| Peak 21 | 0.0527 | 0.0535 | 0.0534 | 0.0542 | 0.0537 | 0.0540 | 0.98 |
| Peak 22 | 0.0300 | 0.0308 | 0.0301 | 0.0316 | 0.0308 | 0.0308 | 1.89 |

**Stability**

TABLE S3-5 The relative retention time of the stability of KHJ

| Number | 0 h | 2 h | 4 h | 6 h | 8 h | 12 h | 24 h | RSD/% |
| --- | --- | --- | --- | --- | --- | --- | --- | --- |
| Peak 1 | 0.1538 | 0.1543 | 0.1545 | 0.1546 | 0.1545 | 0.1546 | 0.1545 | 0.18 |
| Peak 2 | 0.1764 | 0.1771 | 0.1776 | 0.1779 | 0.1780 | 0.1783 | 0.1783 | 0.39 |
| Peak 3 | 0.2248 | 0.2250 | 0.2255 | 0.2255 | 0.2260 | 0.2259 | 0.2259 | 0.21 |
| Peak 4 | 0.3378 | 0.3343 | 0.3336 | 0.3328 | 0.3328 | 0.3321 | 0.3317 | 0.62 |
| Peak 5 | 0.3589 | 0.3575 | 0.3575 | 0.3569 | 0.3579 | 0.3568 | 0.3567 | 0.22 |
| Peak 6 | 0.6252 | 0.6229 | 0.6217 | 0.6202 | 0.6206 | 0.6190 | 0.6186 | 0.37 |
| Peak 7 | 0.7194 | 0.7189 | 0.7195 | 0.7190 | 0.7202 | 0.7196 | 0.7200 | 0.07 |
| Peak 8 | 0.7755 | 0.7740 | 0.7739 | 0.7731 | 0.7737 | 0.7728 | 0.7733 | 0.11 |
| Peak 9 | 0.8061 | 0.8041 | 0.8041 | 0.8029 | 0.8040 | 0.8027 | 0.8033 | 0.14 |
| Peak 10 | 0.8273 | 0.8261 | 0.8262 | 0.8254 | 0.8260 | 0.8252 | 0.8257 | 0.08 |
| Peak 11 | 1.0000 | 1.0000 | 1.0000 | 1.0000 | 1.0000 | 1.0000 | 1.0000 | 0.00 |
| Peak 12 | 1.0479 | 1.0468 | 1.0468 | 1.0471 | 1.0475 | 1.0470 | 1.0475 | 0.04 |
| Peak 13 | 1.1424 | 1.1426 | 1.1430 | 1.1434 | 1.1430 | 1.1433 | 1.1431 | 0.03 |
| Peak 14 | 1.5713 | 1.5731 | 1.5750 | 1.5749 | 1.5739 | 1.5749 | 1.5751 | 0.09 |
| Peak 15 | 1.6496 | 1.6514 | 1.6535 | 1.6533 | 1.6522 | 1.6532 | 1.6535 | 0.09 |
| Peak 16 | 1.6670 | 1.6688 | 1.6700 | 1.6710 | 1.6700 | 1.6710 | 1.6713 | 0.09 |
| Peak 17 | 1.7600 | 1.7626 | 1.7650 | 1.7649 | 1.7638 | 1.7652 | 1.7651 | 0.11 |
| Peak 18 | 1.7765 | 1.7792 | 1.7816 | 1.7816 | 1.7806 | 1.7820 | 1.7822 | 0.12 |
| Peak 19 | 1.8487 | 1.8512 | 1.8538 | 1.8537 | 1.8526 | 1.8541 | 1.8543 | 0.11 |
| Peak 20 | 1.8573 | 1.8601 | 1.8626 | 1.8626 | 1.8616 | 1.8633 | 1.8633 | 0.12 |
| Peak 21 | 1.9371 | 1.9401 | 1.9426 | 1.9427 | 1.9416 | 1.9430 | 1.9430 | 0.11 |
| Peak 22 | 2.2509 | 2.2537 | 2.2413 | 2.2569 | 2.2554 | 2.2565 | 2.2571 | 0.25 |

TABLE S3-6 The relative peak area of the stability of KHJ

| Number | 0 h | 2 h | 4 h | 6 h | 8 h | 12 h | 24 h | RSD/% |
| --- | --- | --- | --- | --- | --- | --- | --- | --- |
| Peak 1 | 0.0179 | 0.0182 | 0.0186 | 0.0187 | 0.0189 | 0.0191 | 0.0193 | 2.64 |
| Peak 2 | 0.1544 | 0.1533 | 0.1533 | 0.1529 | 0.1535 | 0.1534 | 0.1536 | 0.30 |
| Peak 3 | 0.0122 | 0.0114 | 0.0114 | 0.0114 | 0.0115 | 0.0114 | 0.0113 | 2.67 |
| Peak 4 | 0.0787 | 0.0783 | 0.0785 | 0.0785 | 0.0784 | 0.0786 | 0.0786 | 0.17 |
| Peak 5 | 0.2604 | 0.2590 | 0.2587 | 0.2579 | 0.2585 | 0.2592 | 0.2587 | 0.30 |
| Peak 6 | 0.0414 | 0.0412 | 0.0435 | 0.0417 | 0.0439 | 0.0438 | 0.0439 | 2.96 |
| Peak 7 | 0.2261 | 0.2254 | 0.2254 | 0.2246 | 0.2252 | 0.2245 | 0.2254 | 0.24 |
| Peak 8 | 0.0147 | 0.0151 | 0.0150 | 0.0150 | 0.0155 | 0.0152 | 0.0152 | 1.62 |
| Peak 9 | 0.0230 | 0.0226 | 0.0218 | 0.0219 | 0.0232 | 0.0231 | 0.0220 | 2.69 |
| Peak 10 | 0.0299 | 0.0295 | 0.0295 | 0.0295 | 0.0299 | 0.0299 | 0.0296 | 0.69 |
| Peak 11 | 1.0000 | 1.0000 | 1.0000 | 1.0000 | 1.0000 | 1.0000 | 1.0000 | 0.00 |
| Peak 12 | 0.0135 | 0.0138 | 0.0140 | 0.0135 | 0.0135 | 0.0137 | 0.0137 | 1.38 |
| Peak 13 | 0.0222 | 0.0221 | 0.0220 | 0.0221 | 0.0219 | 0.0216 | 0.0208 | 2.24 |
| Peak 14 | 0.0295 | 0.0289 | 0.0291 | 0.0283 | 0.0283 | 0.0285 | 0.0296 | 1.88 |
| Peak 15 | 0.0457 | 0.0435 | 0.0460 | 0.0449 | 0.0459 | 0.0453 | 0.0468 | 2.29 |
| Peak 16 | 0.0149 | 0.0143 | 0.0144 | 0.0144 | 0.0143 | 0.0144 | 0.0146 | 1.48 |
| Peak 17 | 0.0121 | 0.0111 | 0.0112 | 0.0114 | 0.0112 | 0.0113 | 0.0113 | 2.95 |
| Peak 18 | 0.0105 | 0.0104 | 0.0105 | 0.0106 | 0.0105 | 0.0108 | 0.0103 | 1.50 |
| Peak 19 | 0.0067 | 0.0067 | 0.0065 | 0.0066 | 0.0068 | 0.0069 | 0.0069 | 2.22 |
| Peak 20 | 0.0092 | 0.0097 | 0.0095 | 0.0094 | 0.0095 | 0.0094 | 0.0093 | 1.70 |
| Peak 21 | 0.0540 | 0.0538 | 0.0536 | 0.0536 | 0.0536 | 0.0526 | 0.0545 | 1.07 |
| Peak 22 | 0.0274 | 0.0273 | 0.0273 | 0.0271 | 0.0273 | 0.0272 | 0.0287 | 2.00 |

**5 Chemical composition content determination method validation**

##### Precision

TABLE S4-1 Precision test results of each component

| Number | Matrine | Oxymatrine | Bergenin | Macaine |
| --- | --- | --- | --- | --- |
| 1 | 631.5 | 337.1 | 6830.8 | 217.5 |
| 2 | 627 | 343.2 | 6813.9 | 217.3 |
| 3 | 622.7 | 341.4 | 6799 | 216.2 |
| 4 | 624.2 | 341.5 | 6789 | 216.1 |
| 5 | 621.5 | 337.2 | 6784.1 | 215.7 |
| 6 | 626.1 | 338.8 | 6821.4 | 216.5 |
| average value | 625.5 | 339.9 | 6806.4 | 216.6 |
| RSD/% | 0.57 | 0.74 | 0.27 | 0.33 |

**Stability**

TABLE S4-2 Stability test results of each component

| Time | Matrine | Oxymatrine | Bergenin | Macaine |
| --- | --- | --- | --- | --- |
| 0 h | 619.4 | 304.3 | 7005.3 | 214.7 |
| 2 h | 607.2 | 299.9 | 7056.6 | 215.6 |
| 4 h | 618.9 | 304.1 | 7066.5 | 217.6 |
| 6 h | 623.6 | 308.6 | 7067.1 | 217.0 |
| 8 h | 625.6 | 316.8 | 7099.9 | 218.0 |
| 12 h | 595.0 | 300.9 | 7129.3 | 216.6 |
| 24 h | 607.2 | 301.9 | 7016.3 | 217.6 |
| average value | 613.8 | 305.2 | 7063.0 | 216.7 |
| RSD/% | 1.80 | 1.92 | 0.62 | 0.55 |

##### Repeatability

TABLE S4-3 Repeatability content test results of each component（mg/g）

| Number | Matrine | Oxymatrine | Bergenin | Macaine |
| --- | --- | --- | --- | --- |
| 1 | 4.5814 | 2.4875 | 8.6400 | 0.3731 |
| 2 | 4.6257 | 2.4814 | 8.6967 | 0.3899 |
| 3 | 4.6687 | 2.4103 | 8.6833 | 0.3646 |
| 4 | 4.7820 | 2.4545 | 8.8540 | 0.3839 |
| 5 | 4.8037 | 2.5145 | 8.8715 | 0.3747 |
| 6 | 4.7901 | 2.5225 | 8.8817 | 0.3841 |
| average value | 4.7086 | 2.4785 | 8.7712 | 0.3784 |
| RSD/% | 2.03 | 1.67 | 1.24 | 2.44 |

#### Recovery rate

TABLE S4-4 Sample recovery test for each component（n=6）

| Compound | Original  quantity（mg） | Quantity  added（mg） | Measured  quantity（mg） | Recovery  (%) | Average recovery rate(%) | RSD  （%） |
| --- | --- | --- | --- | --- | --- | --- |
| Matrine | 0.2354 | 0.2360 | 0.4740 | 101.10 | 100.57 | 1.42 |
|  | 0.2354 | 0.2360 | 0.4749 | 101.48 |  |  |
|  | 0.2354 | 0.2360 | 0.4686 | 98.81 |  |  |
|  | 0.2354 | 0.2360 | 0.4757 | 101.82 |  |  |
|  | 0.2354 | 0.2360 | 0.4750 | 101.53 |  |  |
|  | 0.2354 | 0.2360 | 0.4683 | 98.69 |  |  |
| Oxymatrine | 0.1239 | 0.1243 | 0.2457 | 97.99 | 97.43 | 2.24 |
|  | 0.1239 | 0.1243 | 0.2452 | 97.59 |  |  |
|  | 0.1239 | 0.1243 | 0.2491 | 100.72 |  |  |
|  | 0.1239 | 0.1243 | 0.2456 | 97.91 |  |  |
|  | 0.1239 | 0.1243 | 0.2435 | 96.22 |  |  |
|  | 0.1239 | 0.1243 | 0.2409 | 94.13 |  |  |
| Bergenin | 0.4386 | 0.4386 | 0.8872 | 102.28 | 101.57 | 0.59 |
|  | 0.4386 | 0.4386 | 0.8835 | 101.44 |  |  |
|  | 0.4386 | 0.4386 | 0.8816 | 101.00 |  |  |
|  | 0.4386 | 0.4386 | 0.8816 | 101.00 |  |  |
|  | 0.4386 | 0.4386 | 0.8874 | 102.33 |  |  |
|  | 0.4386 | 0.4386 | 0.8831 | 101.35 |  |  |
| Macaine | 0.01892 | 0.01881 | 0.03834 | 103.24 | 102.76 | 0.96 |
|  | 0.01892 | 0.01881 | 0.03825 | 102.76 |  |  |
|  | 0.01892 | 0.01881 | 0.03853 | 104.25 |  |  |
|  | 0.01892 | 0.01881 | 0.03822 | 102.60 |  |  |
|  | 0.01892 | 0.01881 | 0.03796 | 101.22 |  |  |
|  | 0.01892 | 0.01881 | 0.03820 | 102.50 |  |  |

TABLE S4-5 The calibration curve equations, r, and the linearity range of four analytes

| Compound | Calibration curve equation | | r | Calibration curve equation/μg |
| --- | --- | --- | --- | --- |
| Matrine | | *Y* = 668.20*X*+4.32 | 1.0000 | 0.1424~1.424 |
| Oxymatrine | | *Y* = 691.56*X*+3.12 | 1.0000 | 0.0579~0.579 |
| Bergenin | | *Y* = 4441.90*X*+174.23 | 0.9999 | 0.3350~3.350 |
| Macaine | | *Y* = 3101.80*X*+3.67 | 0.9996 | 0.01414~0.1414 |

TABLE S5 HPLC Fingerprint Profile Compound Identification Information

| Number | Retention time(min) | Compound Name | Method of Identification | Molecular formula | Molecular weight |
| --- | --- | --- | --- | --- | --- |
| 1 | 7.828 | Matrine | Reference Substance | C_15_H_24_N_2_O | 248.36 |
| 2 | 14.834 | Oxymatrine | Reference Substance | C_15_H_24_N_2_O_2_ | 264.36 |
| 3 | 23.902 | Bergenin | Reference Substance | C_14_H_16_O_9_ | 328.27 |
| 4 | 53.643 | Macaine | Reference Substance | C_16_H_12_O_5_ | 284.26 |
